# Targeting SUMOylation in ovarian cancer: Sensitivity, resistance, and the role of MYC

**DOI:** 10.1016/j.isci.2025.112555

**Published:** 2025-04-29

**Authors:** Samantha Littler, Bethany M. Barnes, Rhys Owen, Louisa Nelson, Anthony Tighe, I-Hsuan Lin, Hugh C. Osborne, Christine K. Schmidt, Joanne C. McGrail, Stephen S. Taylor

**Affiliations:** 1 Division of Cancer Sciences, School of Medical Sciences, Faculty of Biology, Medicine and Health, University of Manchester, Manchester Cancer Research Centre, 555 Wilmslow Road, Manchester M20 4GJ, UK; 2 Bioinformatics Core Facility, Faculty of Biology, Medicine and Health, University of Manchester, Michael Smith Building, Dover Street, Manchester M13 9PT, UK

**Keywords:** Molecular biology, Cancer, Transcriptomics

## Abstract

Cells overexpressing MYC depend on SUMOylation for survival and cell division. To assess the therapeutic potential of SUMO inhibition, we screened 30 patient-derived ovarian cancer models (OCMs) with the SUMO-activating enzyme inhibitor ML-792. While most were resistant, seven displayed intermediate sensitivity, and a further five were particularly sensitive, with sensitivity accompanied by mitotic errors, polyploidy, apoptosis, and PML body expansion. Resistance was linked to *ABCB1* upregulation, and inhibiting drug efflux sensitized eight resistant OCMs. MYC target genes were enriched in sensitive models, consistent with MYC being a potential driver of response. SUMO inhibition induced an adaptive transcriptional response in resistant cells, but this was attenuated in MYC-overexpressing cells, raising the possibility that transcriptional interference disrupts the homeostatic controls required to buffer the inhibition of SUMO signaling. SUMO sensitivity did not overlap with PARP inhibitor sensitivity, supporting the therapeutic potential of apex SUMO inhibitors to target a subset of homologous-recombination-proficient ovarian cancers.

## Highlights

• SUMO-inhibitor sensitivity of 30 patient-derived ovarian cancer models was defined
• Five super-sensitive and seven sensitive models identified
• MYC target genes are enriched in sensitive models
• ABCB1 drug pump is up-regulated in resistant models

## Graphical abstract

**Figure:**
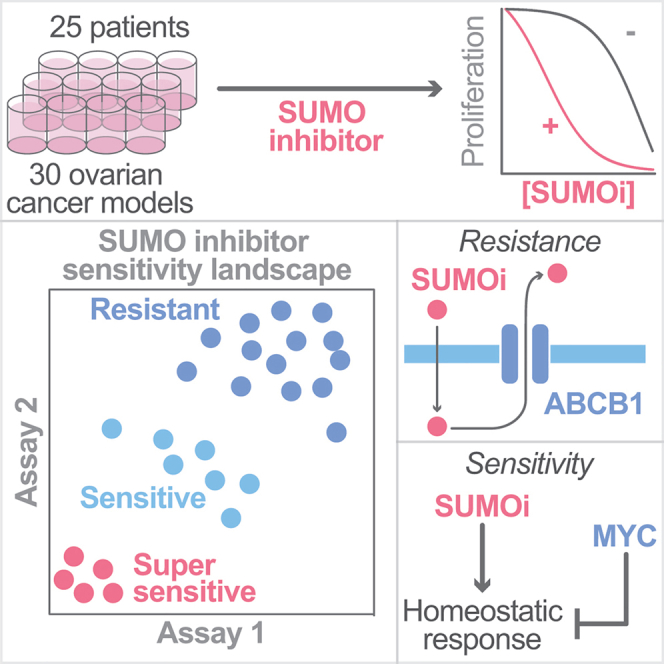

## Introduction

The basic-helix-loop-helix zipper (bHLHZ) transcription factor MYC regulates numerous genes in context-specific manners via cofactor-dependent regulation and transcriptional amplification, in turn regulating numerous biological processes such as metabolism, biogenesis, cell cycle control, and apoptosis.^1^ MYC is also a potent oncogene, capable of driving various cancer hallmarks.^2^

In addition to promoting tumorigenesis, MYC is also required for tumor maintenance; inhibiting MYC in mouse models causes dramatic tumor regression, making it an attractive anti-cancer target.^3^^,^^4^^,^^5^^,^^6^^,^^7^^,^^8^^,^^9^ However, exploiting this therapeutically is challenging because MYC lacks an obvious small-molecule binding pocket, although progress is being made.^10^^,^^11^ In addition, systemic inhibition may yield unacceptable toxicity.^12^ Therefore, indirect approaches are being explored, e.g., focusing on druggable targets that, when inhibited, selectively kill MYC-addicted cells.^13^^,^^14^^,^^15^

In 2012, a screen for MYC-synthetic lethal genes showed that the heterodimeric SUMO E1 activating enzyme is required to tolerate MYC overexpression.^15^ RNAi-mediated inhibition of SAE1 or SAE2, which together form the SUMO E1 enzyme, in human mammary epithelial cells (HMEC) overexpressing MYC, led to abnormal mitoses, polyploidy, and apoptosis. Multiple SAE inhibitors have since been developed, including TAK-981, which has entered clinical trials.^16^ Note, however, that patients are not yet being selected based on MYC status.^17^ Indeed, because MYC serves as a node, integrating multiple upstream and downstream pathways,^18^ MYC status alone may not be a strong predictor of response. Therefore, developing predictive biomarkers capable of selecting patients most likely to benefit from SAE inhibitors will require a better understanding of the relationship between SUMO signaling and MYC overexpression.

Multiple mechanisms could account for why MYC-driven cancer models are dependent on SUMOylation.^15^^,^^19^^,^^20^^,^^21^^,^^22^ One possibility is that the SUMO-MYC interaction affects the expression of a specific subset of genes required for cell division. Indeed, Kessler et al., showed that of 383 genes induced by MYC in HMECs, 86 were repressed upon the inhibition of SAE2.^15^ Twelve of these *MYC switcher genes* encode mitotic factors, including kinetochore components (CENP-A, KNL1), spindle assembly checkpoint regulators (BUB1, BUBR1), centrosome components (ASPM, TPX2), and a cohesion regulator (Sororin). Whether these gene expression changes affect the mitotic proteome is unknown, but they could conceivably disrupt the fidelity of chromosome segregation.

A second possibility is a more broadly ranging effect on transcriptional programming. MYC drives the expression of thousands of genes, including components of the SUMOylation cascade,^19^^,^^21^^,^^22^^,^^23^ but while some studies show that SUMO signaling positively regulates MYC,^15^^,^^24^ other studies suggest that SUMO signaling represses MYC.^25^^,^^26^^,^^27^ Indeed, because SUMOylation inhibits CDK9-dependent transcriptional elongation, MYC and SUMO may act as opposing forces on global gene expression.^28^^,^^29^ Also, multiple transcriptional regulators, including FOXM1b and SMAD3, are modified by SUMO.^30^^,^^31^ However, small-molecule inhibition of SAE only modestly impacts global gene expression,^21^^,^^32^ possibly reflecting an ability of cells to buffer changes in SUMO signaling. This suggests a complex interplay between MYC and SUMO signaling that, if deregulated, could impact multiple transcriptional programs. When coupled with additional oncogenic changes, this could broadly compromise the efficiency of the molecular machines responsible for DNA replication and chromosome segregation, ultimately leading to abnormalities in mitosis.

A third possibility is that MYC-overexpression induces a cell division vulnerability that is buffered by SUMO activity during mitosis. MYC overexpression is synthetically lethal with multiple mitotic regulators,^33^^,^^34^^,^^35^^,^^36^^,^^37^ amplifies drug-induced mitotic perturbations,^38^ and primes the apoptotic machinery that responds to aberrant mitoses.^39^ Numerous mitotic regulators are SUMOylated, including the Aurora kinases, PLK1, BUB1, BUBR1, SGO1, and APC4, and suppressing their modification might exacerbate MYC-induced vulnerabilities.^40^^,^^41^^,^^42^

Thus, the requirement for SUMO signaling to tolerate MYC overexpression could be multifaceted, involving (i) the deregulation of specific subsets of genes; (ii) broad effects on cell cycle transcriptional programs; and/or (iii) direct effects on SUMO substrates involved in mitosis and cell division. To explore these concepts further and the potential of SAE inhibitors to target MYC-addicted cancers, we set out to better understand the relationship between SUMO signaling and MYC overexpression. Here we describe a two-pronged approach. Because MYC is frequently amplified in high-grade serous ovarian cancer (HGSOC),^2^^,^^43^ we screen a panel of ovarian cancer cell lines and 30 patient-derived ovarian cancer models to identify molecular features that correlate with SAE inhibitor sensitivity. In parallel, we deploy a model system whereby MYC can be toggled on and off to further explore the interplay between MYC and SUMOylation.

## Results

### Pharmacological inhibition of SUMO signaling induces cell division failures in MYC-high cells

**Figure 1:**
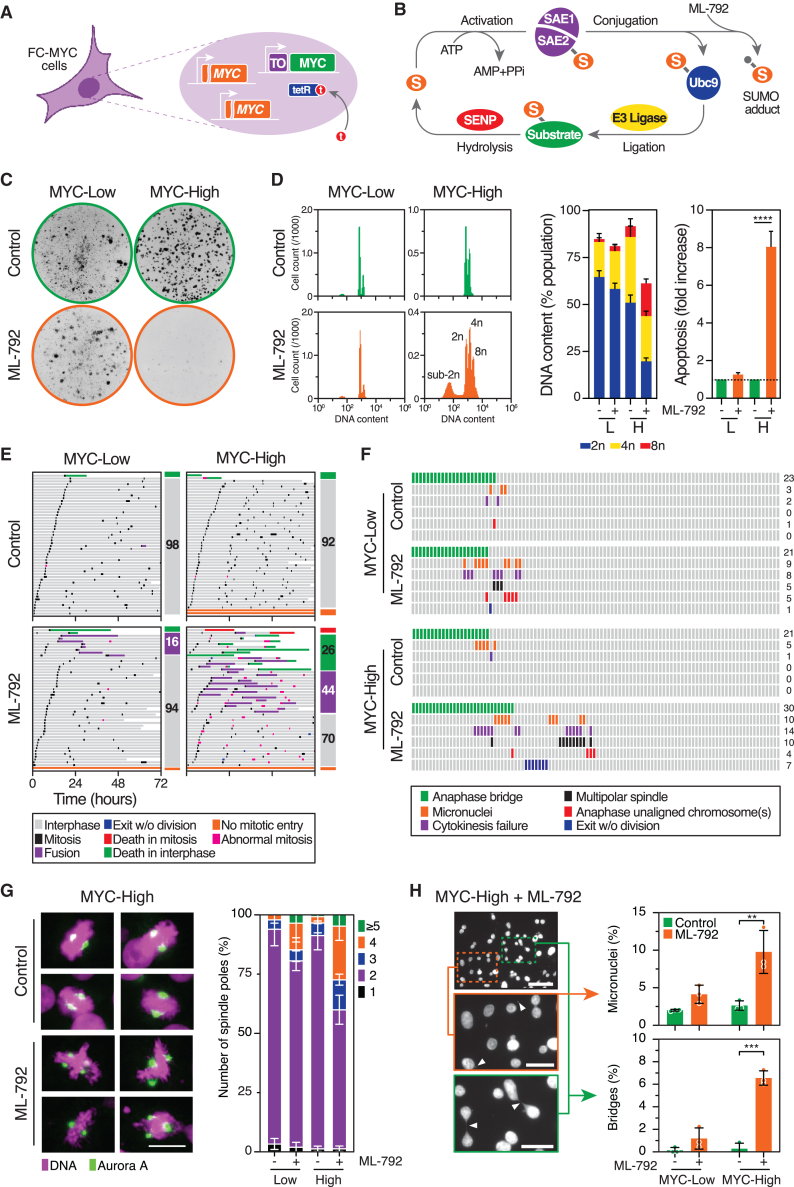
An apex SUMO inhibitor induces cell division failure in MYC-High cells (A) Schematic of FC-MYC cells showing mutated *MYC* alleles (orange) and sequestration of TetR repressors (blue) from the TetO2 operator (purple) by Tetracycline (red) to enable the expression of a *MYC* transgene (green). (B) Canonical SUMO pathway illustrating how SUMO proteins (orange) are activated by the SAE E1 enzyme (purple), transferred to the Ubc9 E2 conjugating enzyme (blue), then ligated to a substrate (green) aided by an E3 ligase (yellow). SUMO is recycled via SENP-mediated hydrolysis (red). ML-792 blocks SUMO signaling via an adduct mechanism that sequesters SUMO proteins. (C) Colony formation assay of FC-MYC cells ± tetracycline, to create MYC-Low and MYC-High states, exposed ± ML-792. (D) DNA content histograms of MYC-Low (L) and MYC-High (H) cells exposed to ML-792 for 72 h, and bar graphs quantifying DNA content and apoptosis (sub-2n) from three biological replicates. two-way ANOVA with Šídák’s multiple comparisons; ∗∗∗∗*p* < 0.0001. (E) Cell fate profiles derived from time-lapse microscopy of MYC-Low/High cells exposed to ML-792, with horizontal bars representing single cells (50 per condition) and colors indicating cell behavior. Numbers in colored boxes show the percentage of cells with the indicated behavior. (F) Time-lapse image analysis of FC-MYC cells expressing GFP-tagged histone, quantifying mitotic abnormalities. Each column represents a single cell with phenotype totals on the right. (G) Immunofluorescence images of mitotic FC-MYC cells exposed to ML-792 for 48 h, stained to detect DNA (purple) and Aurora A (green), and bar graph quantifying the number of spindle poles. Scale bar: 50 μm. Data are mean ± SD from three biological replicates. (H) Fluorescence images of interphase MYC-High cells treated with ML-792 for 48 h were stained to detect DNA, with bar graphs quantifying the percentage of micronuclei and chromatin bridges. Scale bars: 50 μm (*top*) and 20 μm (*middle and bottom*). Data are mean ± SD from three biological replicates with 1000 cells (micronuclei) or 300 cells (bridges) counted per condition. ML-792 was used at 25 nM for all experiments. Two-way ANOVA with Tukey's multiple comparisons ; *∗∗p* < 0.01, *∗∗∗p* < 0.001. See also Figures S1 and S2.

We first deployed FC-MYC cells, where both endogenous *MYC* alleles are inactive due to CRISPR/Cas9-mediated mutation, and MYC function can be toggled on and off using a tetracycline-inducible transgene (Figure 1A).^38^ We chose this RKO-based model in the first instance because, although colon-cancer-derived, it is near-diploid, karyotypically stable^44^ with precisely defined and regulatable MYC status, making it a tractable system to establish experimental parameters including drug exposure concentrations and time-courses. To suppress SUMO signaling, we treated FC-MYC cells with ML-792, an inhibitor of the SUMO SAE E1 activating enzyme.^32^ ML-792 (hereafter SAEi) forms a covalent adduct with SUMO (Figure 1B), catalyzed by SAE, and is one of the most potent SAE inhibitors identified to date, with *in vitro* IC_50_ values in the low nanomolar range. Consistent with RNAi-based experiments,^15^^,^^19^^,^^20^^,^^38^ SAEi inhibited clonogenic potential in MYC-High cells (Figure 1C), accompanied by polyploidy and apoptosis (Figure 1D).

Induction of polyploidy was dose-dependent and became more pronounced over time (Figure S1). The lack of polyploidy in SAEi-treated MYC-Low cells was not due to slower proliferation, as inhibition of Aurora B induced extensive polyploidy in both MYC-Low and MYC-High cells (Figure S2a). Time-lapse microscopy confirmed that, while MYC-High cells cycle faster, both MYC-Low and MYC-High cells entered mitosis in the presence of SAEi (Figure 1E). However, the frequency of abnormal divisions was higher in MYC-High cells, including fusion events many hours after anaphase, indicating a failure of cytokinesis and/or abscission, in turn explaining the subsequent polyploidy.

High-resolution time-lapse microscopy also revealed cytokinesis failures in SAEi-treated cells, as well as multipolar mitoses, anaphases with unaligned chromosomes, anaphase bridges, mitotic exit without chromosome segregation, and the formation of micronuclei, events that were all elevated in MYC-High cells (Figure 1F). This was confirmed by immunofluorescence analysis of fixed cells; multipolar mitoses, anaphase bridges, and micronuclei were markedly increased in MYC-High cells (Figures 1G and 1H).

siRNA-mediated inhibition of Ubc9, the sole known SUMO E2, also led to polyploidy, suggesting that the SAEi effect is via the canonical SUMOylation pathway (Figure S2B). However, an siRNA mini-screen of 14 known SUMO E3 ligases failed to phenocopy the SAEi (Figure S2C). While difficult to draw strong conclusions, this latter observation suggests that the phenotype may not be mediated via a specific SUMO sub-pathway that operates through a single SUMO E3 ligase.

Nevertheless, our observations confirm that the inhibition of SAE, which sits at the apex of the SUMO signaling cascade, leads to abnormal mitoses, polyploidy, and apoptosis, and that these phenotypes are markedly exacerbated by MYC overexpression.^15^^,^^19^^,^^20^^,^^21^^,^^23^^,^^38^

### A subset of ovarian cancer cell lines is sensitive to the inhibition of SUMO signaling

**Figure 2:**
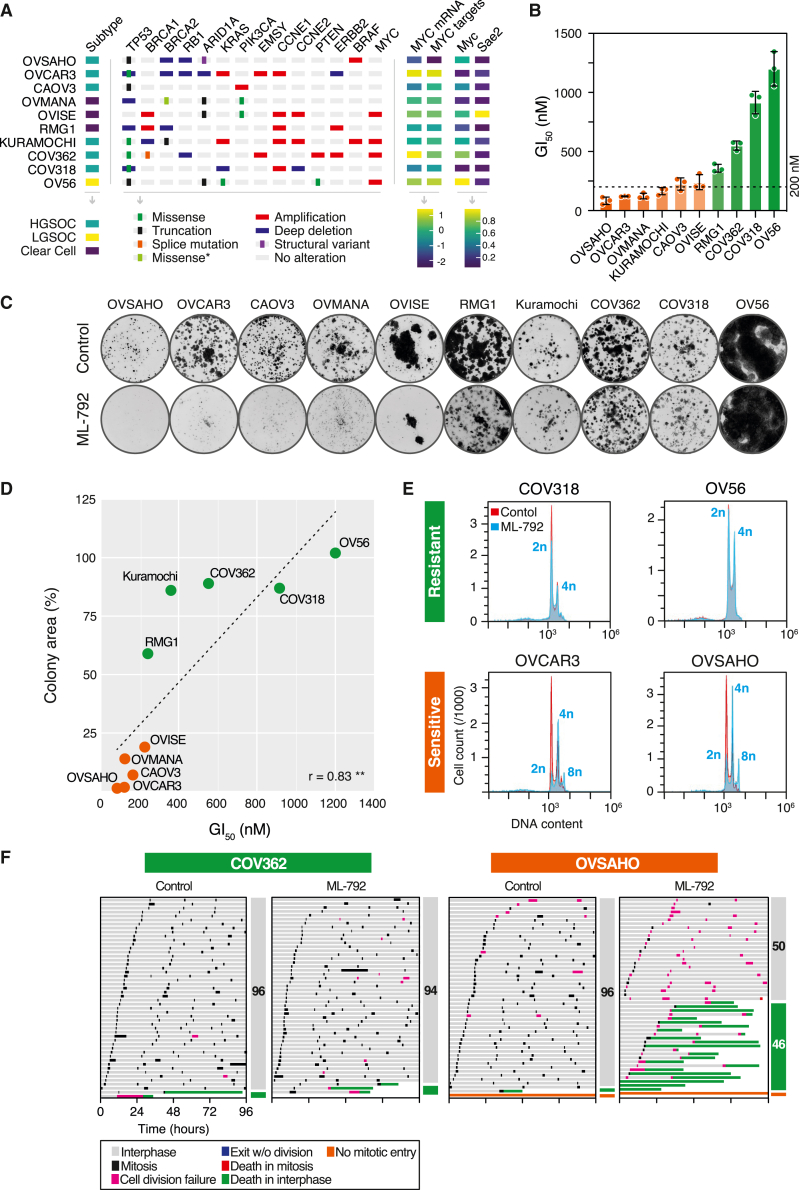
A subset of ovarian cancer cell lines is sensitive to the inhibition of SUMO signaling (A) (*Left*) Mutation profile of selected ovarian cancer cell lines.^45^^,^^46^ ∗ = missense mutation of unknown significance. (*Right*) Expression of MYC and MYC Hallmark Genes V1 based on RNA-sequencing data,^47^ and Myc and Sae2 protein levels determined by LICOR immunoblotting. (B) Bar graph of ML-792 GI_50_ values derived from 96-h proliferation assays, showing mean ± s.e.m from three biological replicates. (C) Representative colony formation assays. (D) *xy* plot of mean GI_50_ values against colony area expressed as a percentage of untreated controls. r provides Pearson correlation value, ∗∗*p* < 0.01. (E) DNA content histograms and (F) cell fate profiles comparing exemplar lines. ML-792 was used at 200 nM for all experiments, and cell fate profiles are as described in Figure 1E. See also Figure S3.

In light of the penetrant anti-clonogenic effect of the SAEi on MYC-High cells, we asked whether ovarian cancer cell lines displayed differential sensitivity to the SAEi. While *MYC* is amplified in many cancers, HGSOC has the highest amplification frequency, at almost 40%.^2^^,^^43^ To do this, we assembled a panel of ten established ovarian cancer cell lines (Figure 2A), six of which bear the hallmarks of HGSOC, namely *TP53* mutations and high chromosome instability scores, three reflect ovarian clear cell carcinoma, and one is more typical of the low-grade serous ovarian subtype.^48^^,^^49^ Importantly, MYC status – as determined by copy number variation, mRNA expression, protein expression, and MYC V1 target gene enrichment – varied across the panel (Figure 2A). SAEi sensitivity was measured using a short-term proliferation assay (Figure 2B) and a long-term clonogenic assay (Figure 2C), revealing five sensitive and five resistant cell lines (Figure 2D). Again, sensitivity correlated with the induction of polyploidy and apoptosis (Figure 2E), and cell fate profiling revealed an increase in abnormal cell divisions and post-mitotic apoptotic events (Figure 2F and S3). However, we observed no obvious correlation between sensitivity and MYC status. This could reflect several factors, for example, the small sample size, cell lines being derived from different disease subtypes, different cell culture media, and network adaptations due to extensive *in vitro* cell culture. To address these issues, we turned our attention to a larger panel of patient-derived ovarian cancer models.^48^^,^^50^^,^^51^^,^^52^

### A subset of patient-derived ovarian cancer models is sensitive to the inhibition of SUMO signaling

**Figure 3:**
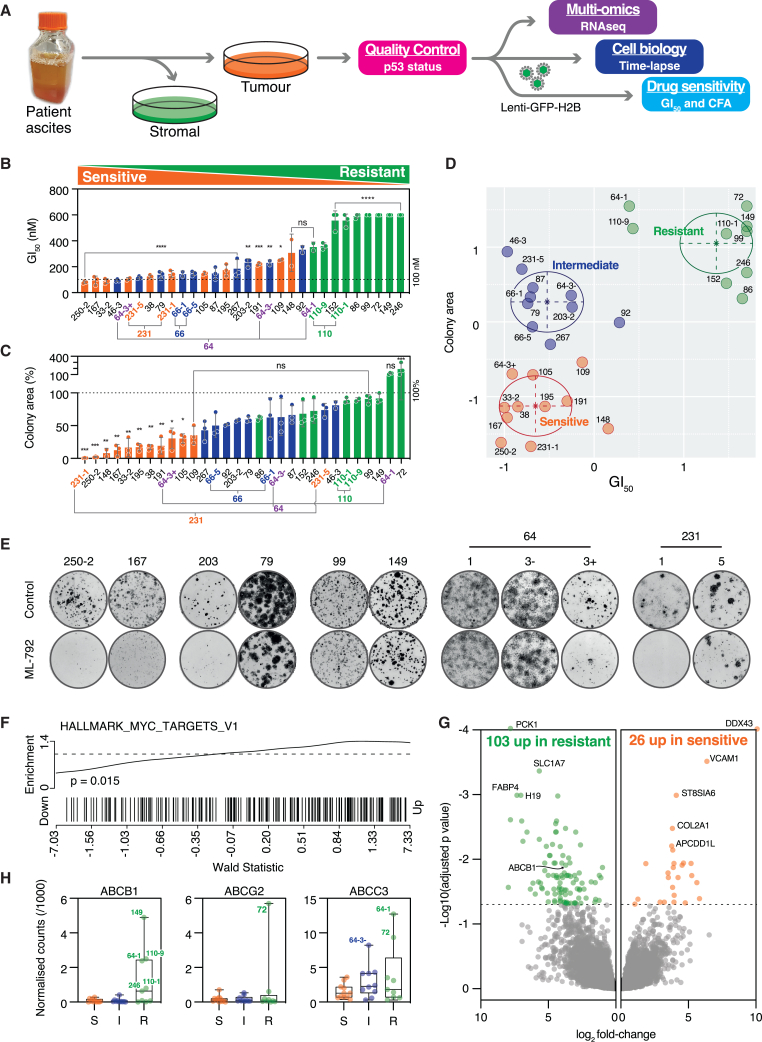
Screen 1: A subset of patient-derived ovarian cancer models is sensitive to the inhibition of SUMO signaling (A) Workflow for generating and characterizing patient-derived ovarian cancer models (OCMs). (B and C) Rank ordered bar graphs showing ML-792 sensitivity of 30 OCMs, based on GI_50_ values determined using a 120-h GFP-H2B-based proliferation assay over a range of drug concentrations (B), and a clonogenic assay performed in the presence of 100 nM ML-792, showing colony formation area expressed as a percentage of untreated cells (C). OCMs derived from the same patient are highlighted, and colors correspond to categories in (D). Bars are mean ± s.e.m from three biological replicates. In (B) and (C), statistical comparisons are to OCMs 110-9 and 149, respectively. ∗*p* < 0.05, ∗∗*p* < 0.01, ∗∗∗*p* < 0.001, ∗∗∗∗*p* < 0.0001, ns: *p* > 0.05. (D) *xy* graph plotting GI_50_ against colony area, highlighting three clusters identified by multivariate mixture modeling, corresponding to OCMs either sensitive (orange), resistant (green), or with intermediate sensitivity (blue). (E) Representative colony formation assays performed at 100 nM ML-792. (F and G) (F) Enrichment of Hallmark MYC targets V1 (*p* = 0.015) and (G) volcano plot shows differentially expressed genes identified by comparing transcriptomes of resistant and sensitive OCMs. (H) Box-and-whisker plots show normalized read counts for indicated *ABC* family members in sensitive (S), intermediate (I), and resistant (R) OCMs. See also Table S1.

To explore correlations between molecular features, clinical data, and drug sensitivity, we have built a living biobank of over 150 patient-derived ovarian cancer models (OCMs).^52^^,^^53^ OCMs display the hallmarks of HGSOC, including p53 alterations, *BRCA1/2* mutations, and diverse karyotypes, consistent with persistent chromosome instability. Importantly, OCMs are cultured in the same media, enabling comparative molecular profiling. OCMs can be studied at early passage, but due to their extensive proliferative potential, are also amenable to longer-term studies, including drug-sensitivity profiling (Figure 3A). To explore the SAEi sensitivity landscape, we selected a panel of 30 OCMs derived from 25 patients (Table S1).

To determine GI_50_ values, the concentration that yields 50% of the maximal inhibition of cell proliferation, OCMs were transduced with a GFP-tagged histone, and nuclear proliferation was measured over 120 h in response to increasing SAEi concentrations. In parallel, clonogenic potential was assessed at 100 nM SAEi via colony formation assay (CFA). This demonstrated that the OCMs display a spectrum of SAEi sensitivity (Figures 3B and 3C). Co-visualising the two assays identified a subset of 11 sensitive and nine resistant OCMs (Figure 3D). Inspecting the colony formation assays confirmed this, e.g., while OCMs 250-2 and 167 were significantly inhibited by the SAEi, OCMs 99 and 149 were largely unaffected (Figure 3E). A third subset of 10 OCMS displayed low GI_50_ values but moderate CFA values, indicating intermediate sensitivity. Note that clonogenic assays were performed at a single SAEi concentration, which may not be high enough to yield a potent effect in the intermediate subset, despite relatively low GI_50_ values.

The panel contains several longitudinal cohorts, i.e., OCMs derived from samples collected from the same patient but at different times during the course of their disease. In two cases, OCMs generated from temporally resolved samples only showed minor differences (patients 66 and 110; Figures 3B and 3C). Two other longitudinal subsets however displayed different SAEi sensitivities. OCMs 64-1 and 64-3 were generated from samples isolated from the same patient 49 days apart, almost 2.5 years after surgery.^52^^,^^53^ OCMs 64-1 and 64-3 contain cells with large, atypical nuclei, negative for PAX8 and EpCAM. However, OCM.64-3 also contains EpCAM-positive cells with small round nuclei positive for PAX8. Exploiting EpCAM status, we separated two distinct subclones; OCMs 64-3+ and 64-3− harbor identical *TP53* mutations (p.V216M) but are positive and negative for EpCAM, respectively. While 64-3− commonly has disomies and trisomies, 64-3+ has numerous monosomies.^52^ Interestingly, 64-1 and 64-3− were relatively SAEi-resistant, while 64-3+ was sensitive (Figures 3B–3E). CFA values for OCMs 231-1 and 231-5 were also markedly different (∼5% and ∼70%, respectively). OCM.231-1 was collected prior to treatment, and 231-5 was collected after the patient was exposed to carboplatin, paclitaxel, and a PARP inhibitor. Because patient 231 was not exposed to a SUMO inhibitor prior to sampling, OCM.231-5’s resistance cannot be due to the selective pressure of suppressed SUMO signaling *in vivo*. Rather, it likely reflects the expansion or sampling of a distinct subclone that is by chance, less dependent on SUMO signaling, or the acquisition of multi-drug resistance, e.g., via a drug efflux mechanism. Nevertheless, these observations clearly demonstrate that patient-derived HGSOC models display a broad spectrum of sensitivity to SAE inhibition, and that different subclones from the same patient can have different SAEi sensitivities.

### Drug efflux inhibition reverses SAE inhibitor resistance

To identify molecular features that correlate with SAEi sensitivity, we analyzed RNA sequencing (RNA-seq) data to identify differentially expressed genes. Importantly, MYC target genes were enriched in SAEi-sensitive OCMs (Figure 3F), consistent with the central hypothesis that MYC overexpressing cells are more sensitive to SAE inhibition. However, we also noted that *ABCB1* was significantly upregulated in resistant OCMs (Figure 3G). *ABCB1* translocations leading to the overexpression of the MDR1/p-glycoprotein drug efflux pump are a significant contributor to chemotherapy resistance in HGSOC.^54^^,^^55^ Therefore, we reasoned that if ML-792 is an MDR1 substrate, then differential SAEi sensitivity could in part be due to multi-drug resistance. And indeed, *ABCB1*, *ABCG2* and *ABCC3* were overexpressed in six SAEi-resistant OCMs (Figure 3H).

**Figure 4:**
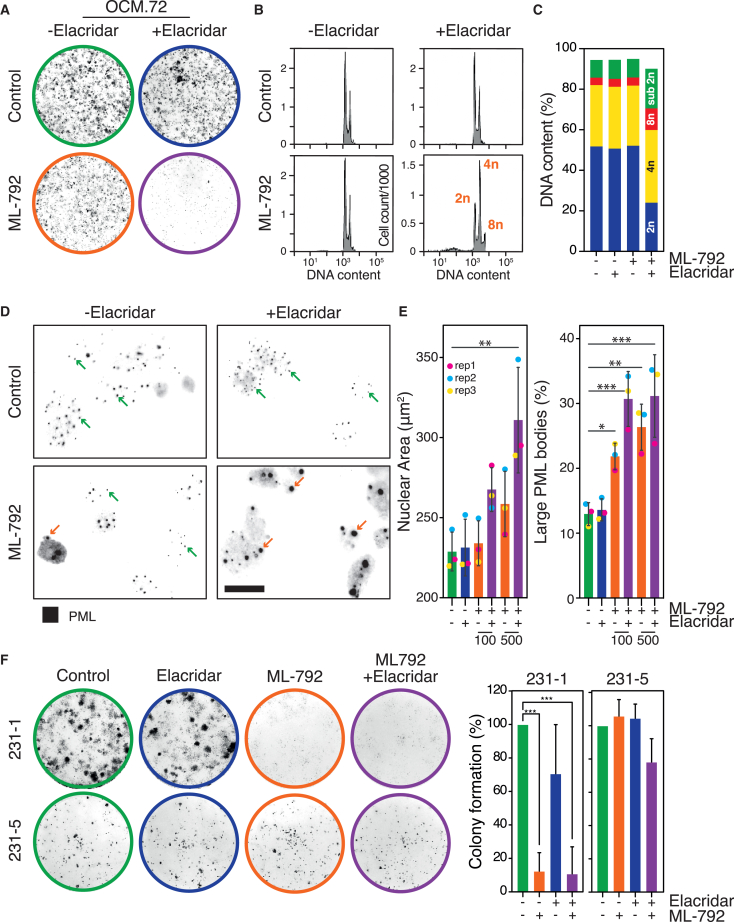
Inhibiting drug efflux can reverse SAE inhibitor resistance (A–E) Analysis of OCM.72 exposed to 500 nM ML-792 and 250 nM elacridar as indicated, showing representative colony formation assay (A); DNA content histograms after 96-h exposure (B); bar graph quantitating cells with DNA contents of 2n (blue), 4n (yellow), 8n (red) or <2n (sub 2n; green) (C); immunofluorescence images of nuclei stained to detect PML bodies highlighting small (green arrows) and large (orange arrows) bodies, scale bar: 20 μm (D); and bar graphs quantitating nuclear area and large PML bodies (E). (F) Analysis of longitudinal OCMs 231-1 and 231-5 exposed to 100 nM ML-792 and 250 nM elacridar as indicated, showing representative colony formation assay and bar graphs quantitating percentage colony area relative to untreated cells. Data are mean ± SD from three biological replicates. One-way ANOVA with Dunnett’s multiple comparisons; ∗*p* < 0.05, ∗∗*p* < 0.01, ∗∗∗*p* < 0.001.

To test whether drug efflux contributes to SAEi resistance, we co-exposed OCM.72 – which overexpresses *ABCG2* – to ML-792 and elacridar, a third-generation efflux inhibitor, which targets ABCB1 and ABCG2.^56^ While the SAEi and elacridar alone had little effect on colony formation, the combination had a dramatic suppressive effect (Figure 4A), which was accompanied by polyploidy and apoptosis (Figures 4B and 4C). Next, we analyzed promyelocytic leukemia (PML) nuclear bodies, which require SAE activity for turnover.^57^ While the SAEi alone had only a modest effect, co-exposure with elacridar significantly increased the number of larger bodies (Figures 4D and 4E). These observations suggest that ML-792 is an efflux substrate and that SAEi resistance can be driven by the upregulation of drug efflux pumps, in line with the identification of *ABCG2* in a recent genome-wide CRISPR screen with ML-792.^58^ Interestingly, elacridar did not sensitize OCM.231-5 to the SAEi (Figure 4F), suggesting that resistance in this case is either driven by a drug pump not targeted by elacridar, or a mechanism that is independent of drug transporters. These observations indicate that the OCM-SAEi sensitivity spectrum is confounded by multi-drug-resistance mechanisms, which mask underlying differences in dependency on MYC or SUMO signaling.

### Re-defining the spectrum of SAE inhibitor sensitivity in the presence of a drug efflux inhibitor

**Figure 5:**
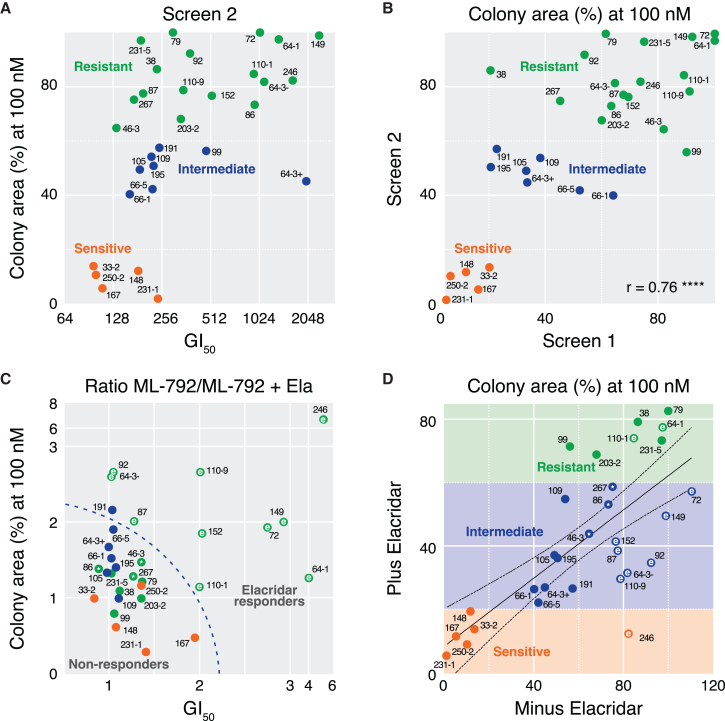
Screen 2: inhibition of drug efflux activity to redefine ML-792 sensitivity (A) *xy* graph plotting GI_50_ values derived from 144-h GFP-H2B-based proliferation assays over a range of drug concentrations, against colony area at 100 nM ML-792 as a percentage of untreated controls. (B and C) (B) *xy* graph plotting percentage colony area at 100 nM ML-792 for Screen 1 versus Screen 2. r provides Pearson correlation value, ∗∗∗∗*p* < 0.0001 (C) *xy* graph plotting the ML-792:ML-792+elacridar (Ela) ratios derived from Screen 2, showing GI_50_ values against percentage colony area at 100 nM ML-792, highlighting elacridar responders. (D) *xy* graph showing percentage colony area at 100 nM ML-792 versus 100 nM ML-792 plus 250 nM elacridar. Line shows a linear regression with 90% confidence bands. The top-10 elacridar responders are highlighted with an “e” and a further three OCMs that move from resistant to intermediate are marked with an “∗”. In (A–D), OCMs are categorized based on colony area at 100 nM ML-792 as sensitive (orange, <20%), intermediate (blue, 20–60%) or resistant (green, >60%). See also Figures S4–S6.

While drug efflux is an important clinical challenge, it represents a confounding factor when correlating SAEi sensitivity with molecular features related to MYC biology and SUMO signaling. To address this, we re-screened the panel of 30 OCMs in the presence and absence of 250 nM elacridar, determining GI_50_ values and clonogenic potential at 100 and additionally at 500 nM SAEi. Again, each OCM was analyzed in three independent experiments (Figures S4A–S4C). Co-visualizing GI_50_ and CFA values in the absence of elacridar again identified sensitive, intermediate, and resistant subsets (Figure 5A). Comparing CFA values demonstrated good congruency with the first screen (Figure 5B), allowing us to further refine the sensitivity categories. We noted three idiosyncrasies and therefore repeated analysis of three OCMs (Figure S4D). Taking the two screens together, we conclude that when exposed to the SAEi alone, 18 OCMs are relatively resistant, five are sensitive and a further seven display intermediate sensitivity (Figure 5B).

To identify elacridar responders, we calculated the (SAEi):(SAEi+elacridar) ratios for both GI_50_ and CFA values (Figure 5C), highlighting 10 SAEi-resistant OCMs that were more sensitive to the SAEi in the presence of elacridar, including OCMs 149 and 246, which overexpress *ABCB1*, and OCM.72 that overexpresses *ABCG2* (Figure 3H). The clonogenic potential of most OCMs was obliterated at 500 nM SAEi, especially in the presence of elacridar (Figure S5), indicating that ABC transporter overexpression has a potent protective effect in a subset of OCMs.

Therefore, to define a sensitivity spectrum, we focused on CFA values at 100 nM SAEi plus elacridar. The 18 OCMs originally considered SAEi-resistant (Figure 5B) included the top-10 elacridar responders, with 1 becoming sensitive (OCM.246) and seven now showing intermediate sensitivity (e.g., OCMs 72, 152 and 110-9) (Figure 5D). Two of the top-10 elacridar responders remained relatively resistant, at least based on the CFA at 100 nM; note that OCM.110-1 was a relatively weak elacridar responder, and the response of OCM.64-1 was driven by the change in GI_50_ value (Figure 5C). Thus, overall screen two finds that when drug efflux is suppressed eight OCMs become sensitive to the SAEi. Three additional OCMs moved from SAEi-resistant to the intermediate range, namely 46-3, 86 and 267; note that these were initially at the “more sensitive” end of the resistant range (Figure 5C), so this change could be due to either weak elacridar responses or variability when assaying complex cell cultures.

Interrogating the CFA images confirmed these conclusions. At the sensitive end of the spectrum, OCMs 231-1, 250-2 and 167 were largely obliterated by 100 nM SAEi (Figure S5 panel i). OCM.246 efficiently formed colonies in 100 nM SAEi and was initially scored as resistant (Figure 5B). However, it was markedly suppressed by the addition of elacridar (Figure S5 panel ii). At the resistant end, 100 nM SAEi had little apparent impact on OCMs 79, 38 and 64-1 (Figure S5 panel iii). OCMs designated as intermediate were typically only marginally affected by 100 nM SAEi but substantially inhibited at 500 nM, either without (e.g., 109, 195), or with elacridar (e.g., 87, 149) (Figure S5 panel iv).

Collectively, these observations indicate that for a third of OCMs screened, SAEi resistance is driven at least in part by drug efflux, confirming that multi-drug resistance is a confounding factor when profiling emerging drugs against a panel of patient-derived HGSOC models. However, by re-screening in the presence of a drug pump inhibitor, we have defined a spectrum of SAEi sensitivity that should better reflect underlying dependencies on SUMO signaling.

### Interrogating gene expression profiles can identify molecular determinants of drug sensitivity

A critical question is whether interrogating molecular features generated by unbiased omics analyses can identify mechanisms that determine OCM drug responses. Having identified the top-10 elacridar responders (Figures 5C and S6A), we used this as a test case and re-interrogated the RNA-seq data. Differential gene expression analysis comparing the 10 elacridar responders with the non-responders yielded only 129 differentially expressed genes (Figure S6A), but importantly, *ABCB1* was significantly upregulated. Thus, interrogating transcriptomes can identify OCM drug-resistance mechanisms, at least when resistance is the result of a dominant, gain-of-function phenotype driven by the upregulation of a single, common gene. Interestingly, *ABCG2* was also identified (Figure S6A), even though only upregulated in a single OCM (Figure 3H), indicating that this approach can identify relatively rare events. Whether this approach can detect loss-of-function phenotypes that yield drug sensitivity or identify more complex vulnerabilities involving redundant networks remains to be seen.

### Ovarian cancer models harbor multiple, non-overlapping vulnerabilities

**Figure 6:**
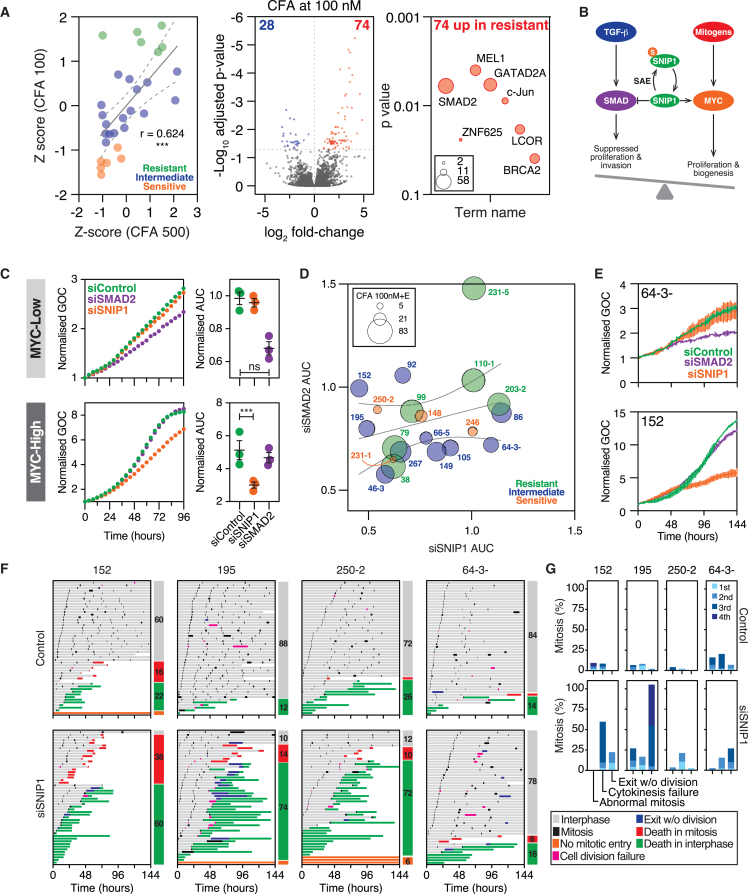
Ovarian cancer models harbor multiple, non-overlapping vulnerabilities (A) (*Left*) *xy* plot of colony formation area (CFA) values converted to z-scores, comparing ML-792-sensitivity at 100 nM versus 500 nM, both in the presence of elacridar. Colors indicate categories described in Figure 5D. Line shows a linear regression with 95% confidence bands. r represents Pearson correlation value, ∗∗∗*p* < 0.001. (*Middle*) Volcano plot showing differentially expressed genes identified using 100 nM ML-792 plus elacridar z-scores as a continuous variable, highlighting 28 downregulated and 74 upregulated genes associated with increasing resistance to ML-792. (*Right*) TRANSFAC analysis of 74 upregulated genes, with symbol size indicating number of genes. (B) Schematic conceptualizing coordination of TGFβ/SMAD and MYC signaling via SNIP1 SUMOylation. (C) (*Left*) Exemplar GFP-H2B-based, 96-h proliferation curves of FC-MYC cells ± tetracycline following siRNA-mediated inhibition of SNIP1 and SMAD2, with green object count (GOC) determined by time-lapse microscopy and normalized to the T_*0*_ value, i.e., when imaging started. (*Right*) Mean area under the curve (AUC) values ±SD from three biological replicates. ∗∗∗*p* < 0.001; ns: *p* > 0.05. (D) *xy* graph plotting proliferation of 20 OCMs following siRNA-mediated inhibition of SNIP1 and SMAD2. Values are AUC derived from 144-h GFP-H2B-based proliferation assays. Bubble size represents the percentage colony formation area (CFA) at 100 nM ML-792 (M) + 250 nM elacridar (E), and colors represent ML-792-sensitivity categories described in Figure 5D. Line shows a linear regression with 95% confidence intervals. Data are mean ± SD for three biological replicates. (E) Exemplar proliferation curves used to generate AUC values in (D), with GOC determined by time-lapse microscopy and normalized to the T_*0*_ value. Mean ± SD for two technical replicates. (F) Cell fate profiles derived from time-lapse microscopy analysis of OCMs indicated, with profiles as described in Figure 1E. (G) Bar graphs quantifying abnormal cell divisions during the 1^st^, 2^nd^, 3^rd,^ or 4^th^ mitosis. See also Figure S6 and Table S3.

Next, we turned our attention back to molecular features that correlate with SAEi sensitivity. To eliminate drug-efflux as a confounding factor, SAEi sensitivity was defined by CFA values in the presence of elacridar, scaled to Z-scores, and deployed as a continuous variable (Figure 6A). The number of differentially expressed genes was again modest, identifying only 102 and 81 genes at 100 and 500 nM SAEi, respectively (Figures 6A and S6B). Importantly, MYC target genes were enriched in SAEi-sensitive OCMs (Figure S6C), consistent with the central hypothesis.

While this gene list was too small for detailed ontology analysis, it was enriched with SMAD2 targets (Figures 6A and S6D). This association of SMAD2 target gene expression with increasing SAEi resistance suggests that SMAD2 signaling might contribute to SAEi resistance. However, initial experiments using the siRNA-mediated inhibition of SMAD2 did not find any influence on SAEi sensitivity (not shown), indicating that SMAD2 is not directly driving resistance. Surveying the literature also led us to SNIP1 (Smad nuclear interacting protein 1), a transcriptional coactivator and repressor regulated by SUMOylation that modulates both the SMAD2 and MYC pathways.^59^^,^^60^^,^^61^ Thus, alongside the enrichment for SMAD2 targets, this literature raised the possibility that the SUMOylation of SNIP1 might coordinate SMAD2 and MYC pathways to modulate G1 cell cycle controls, in turn suggesting that sensitivity to loss of SNIP1 may correlate with SAEi-sensitivity (Figure 6B). To test this, we transfected FC-MYC cells with siRNAs targeting SNIP1 and SMAD2 (Figures 6C and S6E). While siSNIP1 had no effect on proliferation in MYC-Low cells, in MYC-High cells it resulted in a consistent and significant anti-proliferative effect. Therefore, to explore this further, we transfected a panel of 20 OCMs with the siRNAs targeting SNIP1 and SMAD2 and determined proliferation dynamics using time-lapse microscopy. Interestingly, while some OCMs were sensitive to siSMAD2 and/or siSNIP1 in the absence of the SAEi, there was no obvious correlation between sensitivity to siSNIP1, siSMAD2 or the SAEi (Figures 6D, 6E, and S6F).

Exploring siSNIP1-sensitivity in more detail, we observed that OCMs 152, 195 and 250-2 had diminished proliferation with siSNIP1, while OCM.64-3− was relatively resistant (Figures 6E and S6F). Cell fate profiling showed that siSNIP1 in OCMs 152 and 195 resulted in delayed mitoses followed either by death-in-mitosis, or abnormal divisions followed by death-in-interphase (Figures 6F and 6G). Cell death also increased markedly in OCM.250-2, albeit without an obvious delay in mitosis. By contrast, for insensitive OCM.64-3−, while mitosis was modestly affected in the second cell cycle, the vast majority of cells nevertheless divided successfully, and the extent of cell death was largely unaffected. Thus, these observations indicate that in a subset of OCMs, the inhibition of SNIP1 increases cell division abnormalities followed by cell death. Therefore, inhibiting SUMO signaling or SNIP1 results in abnormal mitosis and cell division failure, suggesting both may synergize with underlying chromosomal instability.

Interestingly there was also no correlation between sensitivity to siSMAD2 and siSNIP1 (Figure 6D). One possible explanation for the data in Figure 6D is that these OCMs harbor several different vulnerabilities that when probed exacerbate proliferative deregulation, in turn explaining why different interventions yield a spectrum of OCM sensitivities with little correlation. This idea also draws on our recent experience with replication stress response (RSR) inhibitors. Despite most HGSOC suffering from replication stress^62^^,^^63^ our prior study found that sensitivity to ATR, CHK1, WEE1, and PARG inhibitors was not correlated, indicating RSR-targeting drugs were not interchangeable.^51^ And yet, 15 of the 16 OCMs analyzed were sensitive to a low-dose ATR-CHK1 inhibitor combination. This idea also draws on the observation that deregulating transcriptional regulators E2F1, MYB2L, and FOXM1 in the presence of *TP53* mutation, leads to mitotic errors.^64^ Indeed, HGSOCs can overcome proliferative controls and acquire chromosome instability via multiple mechanisms,^62^^,^^65^^,^^66^ and our observations indicate that this genetic diversity manifests as phenotypic heterogeneity in turn leading to multiple, non-overlapping vulnerabilities.

### Inhibition of SUMO in MYC-Low cells induces a strong adaptive transcriptional response

While the phenotypic heterogeneity exposed above aligns well with the molecular heterogeneity exhibited by HGSOC, it suggests that, in contrast to the *ABCB1* gain-of-function exemplar, comparing transcriptomes from cohorts of distinct OCMs may be less effective at identifying loss-of-function phenotypes involving network-based and/or redundant vulnerabilities. Therefore, to explore whether other proliferative gene expression networks might influence SAEi sensitivity and/or be modulated by SAEi exposure, we returned to the FC-MYC model deployed above and analyzed transcriptomes of MYC-High and MYC-Low cells in the presence and absence of SAEi.

**Figure 7:**
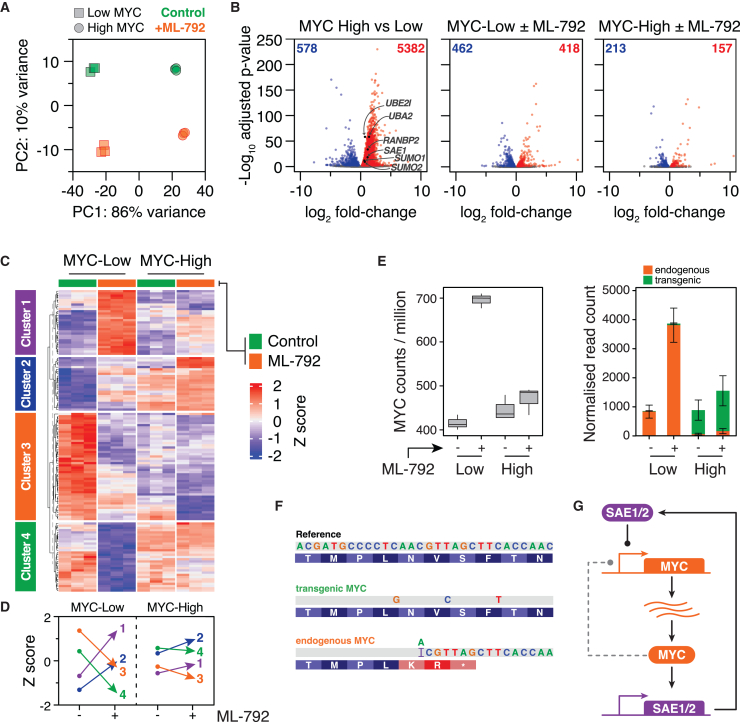
MYC overexpression attenuates an adaptive response induced by the inhibition of SUMO signaling (A) Principal component analysis of RNA sequencing data derived from FC-MYC cells ± tetracycline to create MYC-Low and MYC-High states and exposed to ML-792 for 24 h. The three biological replicates are shown for each condition. (B) Volcano plots show differentially expressed genes comparing the conditions indicated. (C) Heatmap of 132 differentially regulated genes identified by interaction analysis, clustered using K-means. (D) Average z-scores of the four clusters described in (C). (E) (*Left*) Boxplot shows MYC read counts. (*Right*) Normalized MYC read counts distinguishing between endogenous and transgenic mRNA. Values are mean ± SD from three biological replicates. (F and G) (F) Nucleotide and amino acid sequences of *MYC*, showing the three silent point mutations in the transgenic allele, and the mutation introduced at the endogenous alleles by CRISPR/Cas9-mediated gene editing.^38^ (G) Model showing negative feedback loop whereby SUMO signaling inhibits MYC expression. See also Figure S7.

To account for MYC’s transcriptional amplifier function, RNA-seq data were normalized to a set of 166 genes identified as potential MYC-resistant house-keepers in a meta-analysis of published data.^67^ Principal component analysis (PCA) showed that MYC induction correlated with the vast majority of the variation (Figure 7A), leading to 5382 upregulated genes and 578 downregulated genes (Figure 7B). By contrast, SUMO inhibition had a modest effect, accounting for less variation and only 880 gene expression changes in MYC-Low cells and 370 in MYC-High cells (Figure 7B).

Gene set enrichment analysis showed that in the absence of SAEi, MYC induction resulted in an enrichment of MYC target genes (Figure S7A), indicating that analyzing the MYC Hallmark Genes v1 set is a valid proxy for MYC activity. Consistent with MYC’s established roles, pathway analysis identified genes associated with metabolism, biogenesis, and cell cycle processes (Figure S7B). MYC-High transcriptomes were also enriched for SUMOylation genes, including *SUMO1*, *SUMO2*, *SAE1*, *SAE2/UBA2*, *UBC9/UBE2I,* and *RanBP2*, indicating that MYC drives the expression of the SUMOylation machinery (Figures 7B and S7B).^19^^,^^21^^,^^22^^,^^23^ The SAEi also induced MYC target genes in MYC-Low and MYC-High cells, suggesting that SUMO signaling counteracts MYC (Figure S7A). Given that the endogenous *MYC* locus has undergone CRISPR-mediated mutation leading to non-functional transcripts in MYC-Low cells, it is not immediately obvious how SAE inhibition drives MYC target genes in this context. This could reflect leaky transgene expression, or a role for SUMO signaling as a repressor of pro-proliferative transcription,^68^ with MYC targets being regulated by multiple overlapping transcriptional mechanisms.^69^ Indeed, TRANSFAC analysis shows that E2F and MAZ targets were enriched upon SAEi exposure (Figure S7B). Moreover, gene ontology, KEGG, and REACTOME analyses identified TGFβ, FoxO, and SMAD2/3/4 as downregulated in MYC-Low cells exposed to SAEi (Figure S7B), further indicating that the net effect of SUMO signaling is anti-proliferative.

Next, we analyzed the interaction effect that, strikingly, identified 132 genes whose change in expression following SAEi exposure significantly differed between MYC-Low and MYC-High cells, with k-means clustering identifying four blocks of genes with different behaviors (Figure 7C). Cluster 1 genes are very low in MYC-Low cells but increase massively upon SAEi exposure. These genes are also low in MYC-High cells but remain low in response to the inhibition of SUMO signaling. Cluster 2 genes are also very low in the MYC-Low state and increase to intermediate levels upon SAEi exposure (Figure 7C). These genes are already high in MYC-High cells and only modestly increase upon SAE inhibition. Cluster 3 genes are very high in MYC-Low cells and decrease markedly upon SAEi exposure. In MYC-High cells, these genes are initially at low/intermediate levels and decrease only modestly in the SAEi. Finally, Cluster 4 genes are high/intermediate in MYC-Low cells, then massively decrease upon SAEi exposure. While also initially high/intermediate in MYC-High cells, they remain static in the presence of the SAEi. Thus, the overexpression of MYC dramatically attenuates the transcriptional changes induced by the inhibition of SUMO signaling (Figure 7D).

These observations lead to a new proposal; rather than SUMO signaling being required to tolerate MYC overexpression, we suggest that in MYC-Low conditions, SUMO inhibition leads to a strong adaptive response such that the phenotypic consequences are modest. However, the overexpression of MYC markedly attenuates this adaptive response (Figure 7D) such that the inhibition of SUMO signaling is not appropriately buffered, yielding a cell cycle vulnerability that later manifests as an increased frequency of abnormal mitoses and cell divisions (Figure 1).

### SUMO signaling inhibits MYC expression

Strikingly, SAEi exposure elevated MYC transcripts in MYC-Low cells (Figure 7E). Sequence analysis showed that these transcripts harbored the CRISPR mutation and were thus derived from the endogenous *MYC* alleles (Figure 1A), rather than the tet-regulated transgene, and, as such, they cannot generate functional MYC protein (Figure 7F). By contrast, endogenous *MYC* transcripts were only marginally elevated in MYC-High cells exposed to SAEi, although transgenic transcripts were elevated (Figure 7E). One possibility is a feedback loop whereby MYC drives the expression of SUMO signaling, which in turn suppresses *MYC* (Figure 7G). However, because the endogenous *MYC* alleles in FC-MYC RKO cells are defective, this feedback loop is broken, so the transcription of endogenous *MYC* is unrestrained, leading to the accumulation of defective transcripts (Figure S7C). Note that the tet-induced expression of MYC suppressed the accumulation of these defective transcripts (Figure 7E), consistent with the existence of SUMO-independent negative autoregulation.^70^^,^^71^^,^^72^ This supports a more general model whereby MYC drives the expression of the SUMOylation machinery, while SUMO signaling counterbalances MYC, in part by directly repressing *MYC* itself (Figure 7G).

## Discussion

Here, we show that cells engineered to overexpress MYC are sensitive to an apex SUMO inhibitor. This interaction was first identified in 2012,^15^ and the high-level phenotype, namely abnormal mitosis, polyploidy, and apoptosis, has been observed independently by us and others.^15^^,^^19^^,^^21^^,^^38^ But the underlying mechanism remains to be delineated, suggesting that perhaps the current conceptual framework needs revisiting. The original notion drew parallels with synthetic lethality, suggesting that SUMO signaling is required to tolerate MYC overexpression.^15^ Microarray analysis revealed a transcriptional subprogram of SUMOylation-dependent MYC switcher (SMS) genes, which flipped from active to repressed upon SAE inhibition in MYC overexpressers, and a subset of these switchers encoded mitotic regulators, providing a rationale for the polyploidy phenotype. Our RNA-seq-based analysis shows that the MYC–SUMO interaction is more complex, revealing four clusters of genes modulated differently. However, while a subset within Cluster 3 is downregulated in MYC-High cells upon SAEi exposure, there is no overlap with the original SMS genes, and it does not include mitotic regulators. Moreover, this subset did not flip; the SAEi also induced downregulation in MYC-Low cells.

Our analysis points to a fundamentally different possibility, whereby in the absence of MYC overexpression, the inhibition of SUMO signaling leads to a strong adaptive response, with some genes being markedly upregulated, others downregulated. However, the overexpression of MYC dramatically attenuates this response; the expression levels move in the same direction, but the magnitude of the change is substantially reduced. It is possible, therefore, that this adaptive response allows cells to buffer the loss of SUMO signaling, thus maintaining proteostasis of networks required to coordinate cell cycle progression, DNA replication, chromosome segregation, and cytokinesis. In turn, attenuating this response might disrupt cell cycle networks, leading to replication and/or segregation errors, driving cell division failure and polyploidy. The penetrance of this effect may be exacerbated because MYC overexpression is sufficient to induce cell division vulnerabilities.^33^^,^^34^^,^^35^^,^^36^^,^^37^^,^^38^ Indeed, because successful cell division requires the fidelity of numerous upstream processes, vulnerabilities at many points in the cell cycle could ultimately manifest in mitotic failure. Thus, the MYC-SUMO effect may not be due to the disruption of a specific gene expression sub-program or the inhibition of specific SUMO sub-pathway operating in mitosis, but rather the inability to mount an adaptive response to suppressed SUMO signaling.

How might MYC overexpression compromise this adaptive response? While the inhibition of SUMO signaling only modulates a few hundred genes, the overexpression of MYC modulates thousands. One possibility, therefore, is that upon loss of SUMO signaling, specific transcription factors and coactivator complexes are recruited to the promoters of appropriate target genes to elicit the necessary response. However, by increasing the number of active genes by ∼7-fold, MYC overexpression might sequester a subset of these transcriptional regulators to a much larger number of promoters, in turn depleting them from the genes required to mount the adaptive response to the inhibition of SUMO signaling. This is reminiscent of cofactor squelching, or transcriptional interference, whereby an active transcription factor recruits coactivators to promoters of its target genes, thereby limiting the availability of these factors to other transcription factors, resulting in the repression of their target genes.^73^ Conceptualized almost 40 years ago, and well characterized in the context of nuclear receptors and NFkB signaling, the exact mechanisms and physiological relevance of cofactor squelching have remained controversial. Interestingly, in a mouse model of prostate cancer, overexpression of MYC dampened the androgen receptor transcriptional program via a mechanism involving cofactor redistribution.^74^ Whether the attenuation of the adaptive response triggered by SUMO inhibition does involve a MYC-induced cofactor squelching mechanism remains to be seen, and exploring this concept further in the FC-MYC model system will be an important next step.

Our observations support previous studies showing that MYC upregulates the SUMOylation machinery.^19^^,^^21^^,^^22^^,^^23^ An added layer of complexity is the ability of SUMO signaling to negatively regulate MYC. Negative autoregulation of MYC is well established, and with various mechanisms proposed, including the suppression of transcription initiation,^70^^,^^71^^,^^72^ induction of translation-blocking microRNAs,^75^ and activation of ubiquitination-mediated degradation pathways.^76^ Our study supports the notion that SUMO signaling directly suppresses MYC expression. This is consistent with previous studies showing that inhibiting the SUMO E2 Ubc9 upregulates MYC in a CDK9-dependent manner.^27^ MYC itself is subject to SUMO conjugation via the SUMO E3 ligase PIAS1, which enhances subsequent ubiquitination and degradation.^25^ Thus, SUMO signaling counteracts MYC at multiple levels. Indeed, while MYC drives proliferation and biogenesis, SUMO signaling opposes this. SUMOylation inhibits global Pol-II activity by suppressing CDK9-dependent transcriptional elongation,^28^ and limits Pol I-dependent rDNA transcription, suppressing ribosome biogenesis.^27^

These opposing activities, linked via a negative feedback loop, may constitute a homeostatic control mechanism, coupling pro- and anti-proliferative signals with biogenesis pathways. While oncogenic MYC activity may favor a pro-proliferative state, homeostatic control may nonetheless be maintained by active SUMO signaling. Similarly, in the absence of overexpressed MYC, SUMO blockade is buffered by other aspects of MYC's negative autoregulation. However, SUMO blockade in the presence of overactive MYC may sufficiently disrupt homeostasis to unleash MYC activity and de-repress anti-proliferative controls, resulting in cells “*bulldozing”* their way through the cell cycle, increasing the risk of DNA replication and chromosome segregation errors, ultimately manifesting as cell division failure and polyploidy. However, even in a well-defined model system, this framework is likely too simplistic; an elegant live-cell imaging study recently showed that MYC expression is pulsatile and highly heterogeneous.^72^ This dynamic behavior originates from transcriptional regulation rather than protein turnover, can be modulated by upstream pathways, and in turn can influence cell fate. Thus, parameters that modulate MYC dynamics may influence a cell’s ability to buffer SUMO blockade, and vice versa, inhibiting SUMO signaling may modulate MYC dynamics in turn influencing MYC function. Exploring the SAEi effect on MYC dynamics will be an important next step.

Extrapolating observations derived from karyotypically stable model systems to more complex, patient-derived HGSOC models, which display profound karyotype heterogeneity due to persistent chromosome instability, is challenging. Although the correlation is modest, MYC target genes are enriched in SAEi-sensitive OCMs, consistent with the central hypothesis that the overexpression of MYC exposes the survival requirement for SUMO signaling. Whether the inhibition of SUMOylation drives an adaptive response in OCMs, and whether this is modulated by MYC-driven cofactor squelching and/or MYC dynamics, are unknowns. The challenge arises because HGSOC displays profound molecular heterogeneity^77^^,^^78^ that is captured by the OCMs, which in turn display profound phenotypic heterogeneity.^52^ Thus, it is possible that multiple mechanisms may influence OCM SAEi sensitivity, even within individual OCMs.

It is striking that when comparing SAEi-sensitive versus resistant OCMs, the number of differentially expressed genes was low. Despite this, there was an enrichment for SMAD2 target genes in SAEi-resistant OCMs. Taking this into account with the homeostatic control concept described above, and the available literature, we conceptualized a network linking pro-proliferative MYC signaling with anti-proliferative SMAD signaling via the SUMOylation of SNIP1.^59^^,^^60^^,^^61^ While inhibiting SMAD2 alone does not appear to overcome SAEi resistance, we did uncover three non-overlapping vulnerabilities whereby subsets of OCMs were sensitive to the inhibition of SMAD2 or SNIP1 or SUMO signaling, with little correlation between the three interventions. And in the case of inhibiting SUMO signaling or SNIP1, probing the vulnerabilities results in abnormal mitosis and cell division failure, suggesting that these vulnerabilities may synergize with underlying chromosomal instability mechanisms to suppress clonogenic potential. On the one hand, this suggests that the transcription networks regulating G1 cell cycle controls could be a rich source of therapeutic targets. On the other hand, it also suggests that considerable complexity exists and that delineating the underlying biology of these different vulnerabilities will be challenging. However, because the OCMs capture the broad spectrum of molecular features displayed by HGSOC,^52^^,^^53^ they provide a tractable platform to describe the genetic and pharmacological vulnerability landscape of HGSOC.

The SUMOylation inhibitor TAK-981 has entered early phase clinical trials, focusing on patients with advanced or metastatic solid tumors (non-squamous non-small cell lung cancer, cervical cancer, microsatellite stable colorectal cancer) or relapsed/refractory multiple myeloma.^17^ TAK-981 is also an apex SUMO inhibitor, targeting the SAE1/2 heterodimer via the same mechanism as ML-792.^16^ Although slightly less potent *in vitro*, TAK-981 shows more durable responses in pre-clinical tumor models. It also induces chromosome segregation errors and polyploidy in human pancreatic cancer cell lines.^42^ Our results show that a subset of patient-derived HGSOC models is sensitive to an apex SUMO inhibitor of this class. When exposed to ML-792 alone, while 18 of the 30 OCMs screened were largely resistant, five were particularly sensitive, and a further seven showed intermediate sensitivity. Of the 12 sensitive/intermediate OCMs, only two are sensitive to the PARP inhibitor Olaparib, while seven are resistant, and three have not yet been tested (to be described elsewhere). This suggests that SUMO inhibitors may have the potential to target HGSOC independent of homologous recombination (HR) status. Note that while PARP inhibitors are highly effective in cases of HR-deficient disease, ∼60% of HGSOCs are HR-proficient,^62^^,^^79^^,^^80^ and therefore unlikely to respond to a PARP inhibitor. Because there are currently few treatment options for this patient subgroup, evaluating SUMOylation inhibitors in HR-proficient HGSOC should be a priority.

To facilitate the testing of SAE inhibitors for HGSOC, identifying tumors that are critically dependent on SUMO signaling will be important. However, at present, we lack the understanding necessary to design predictive biomarkers to guide patient selection. And indeed, the trials evaluating TAK-981, several of which were recently terminated, did not select patients based on molecular features associated with SAEi sensitivity. Because ectopic overexpression of MYC in established cell lines induces profound SAEi-sensitivity, and because MYC target genes are enriched in SAEi-sensitive OCMs, it is tempting to explore MYC overexpression as a predictive biomarker. However, the association between SAEi sensitivity and MYC activity in OCMs is modest, possibly because MYC is considerably distal from the drug target, and/or multiple mechanisms influence SAEi sensitivity. Indeed, while we provide an alternative framework to explain SAEi sensitivity in cells engineered to overexpress MYC, our understanding of what drives SAEi sensitivity in OCMs is still limited. Deepening this understanding will be essential if we are to develop appropriate predictive biomarkers.

### Limitations of the study

This study shows that the overexpression of ABC drug-efflux pumps is a driver of SAEi resistance, with eight OCMs markedly sensitized to the ML-792 by co-exposure to a drug efflux inhibitor. While the use of ML-792 rather than TAK-981 is a limitation of the current analysis, cell-based permeability assays show that TAK-981 and ML-792 have similar efflux ratios.^16^ Because ∼20% of HGSOCs develop chemoresistance via the upregulation of efflux mechanisms,^54^^,^^55^ we anticipate that this will also confer resistance to TAK-981. Therefore, maximizing the potential of this strategy in HGSOC will require either new drugs that are not substrates of efflux pumps or the ability to identify those tumors overexpressing *ABCB1* and other relevant transporters. The idea that an SAEi-induced adaptive response is attenuated by MYC-induced cofactor squelching is based on analyzing RKO-derived FC-MYC cells. Whether these observations apply to MYC-addicted ovarian cancer cells remains to be seen. And finally, while multiple independent experiments have shown that inhibiting SUMO signaling in cell lines engineered to overexpress MYC induces mitotic errors, polyploidy, and apoptosis,^15^^,^^19^^,^^21^^,^^38^ we still lack a mechanistic link to explain the MYC-SUMO interaction.

## Resource availability

### Lead contact

Further information and reagent requests may be directed to Stephen S. Taylor (stephen.taylor@manchester.ac.uk).

### Materials availability

All unique/stable reagents generated in this study will be made available on request, but we may require a payment and/or a completed materials transfer agreement, in particular if there is potential for commercial application.

### Data and code availability

• OCM RNAseq data generated for this study are available from EBML-EBI (EMBL-EBI: E-MTAB-14568). Additional RNAseq data from 27 OCMs deposited previously,^48^^,^^50^^,^^52^ are available from EMBL-EBI (EMBL-EBI: E-MTAB-7223, EMBL-EBI: E-MTAB-10801, EMBL-EBI: E-MTAB-11000). RNAseq from Myc-Low and Myc-High cells following SUMOylation inhibition with ML-792 are available from EBML-EBI (EMBL-EBI: E-MTAB-14654). All RNAseq data are publicly available as of the date of publication. Original images and microscopy data reported in this article, and any additional information required to reanalyze the data, will be shared by the lead contact upon request.
• Original code for analysis of RNAseq is deposited on Zenodo: https://doi.org/10.5281/zenodo.15125928 and is publicly available as of the date of publication.
• Any additional information required to reanalyze the data reported in this article is available from the lead contact upon request.

## Acknowledgments

SL, BMB, LN, AT and HCO are funded by a Cancer Research UK Programme Grant awarded to SST (C1422/A31334); and the 10.13039/501100000265Medical Research Council (MR/X008088/1); with co-funding from the 10.13039/501100000272NIHR Manchester Biomedical Research Center (NIHR203308); and the 10.13039/501100017008Cancer Research UK Manchester Centre (C147/A25254). RO was funded by a 10.13039/100010269Wellcome Trust PhD studentship (102171/B/13/Z). CKS and HCO are funded by the 10.13039/501100000268Biotechnology and Biological Sciences Research Council (BB/N019997/1) and the 10.13039/501100000265Medical Research Council (MR/X008754/1). The views expressed are those of the author(s) and not necessarily those of the 10.13039/501100000272NIHR or the 10.13039/501100000276Department of Health and Social Care.

We thank the MCRC Biobank for sample collection; the Genomic Technologies Core Facility at The University of Manchester for performing the RNA sequencing; and members of the Taylor lab for advice and comments on the article.

## Author contributions

Methodology: SL, BMB, RO, LN, AT, I-HL, and HCO; investigation: SL, BMB, RO, I-HL, and HCO; data curation: SL, BMB, RO, I-HL, and JCM; validation: SL; formal analysis: SL, BMB, RO, I-HL, and SST; conceptualization: SST; funding acquisition: JCM, BMB, CKS, and SST; supervision: CKS and SST; project administration: SL and JCM; resources: SL, LN, and AT; software: BMB; visualization: SL, BMB, and SST; writing original draft: SST; writing (review and editing): SL, BMB, I-HL, JCM, and SST. All authors read and approved the final article.

## Declaration of interests

The authors have no competing interests to declare that are relevant to the content of this article.

## STAR★Methods

### Key resources table

**Table:** 

REAGENT or RESOURCE	SOURCE	IDENTIFIER
**Antibodies**		

Donkey anti-Mouse Cy2	Jackson Immuno Research Laboratories Inc	Cat# 715-225-150; RRID: AB_2340826
Donkey anti-Mouse Cy3	Jackson Immuno Research Laboratories Inc	Cat# 715-165-150; RRID: AB_2340813
Donkey anti-Rabbit Cy2	Jackson Immuno Research Laboratories Inc	Cat# 711-225-152; RRID: AB_2340612
Donkey anti-Rabbit Cy5	Thermo Fisher Scientific	Cat# A10523; RRID: AB_2534032
Goat anti-Mouse IgG (HL) HRP	Invitrogen	Cat# G21234; RRID: AB_2536530
Goat anti-Rabbit IgG (HL) HRP	Merck Millipore	Cat#ABC240; RRID: AB_2722647
Rabbit anti-Sheep IgG (HL) HRP	Invitrogen	Cat# G21040; RRID: AB_2536527
Rabbit anti-Goat IgG (HL) HRP	Invitrogen	Cat# 81-1620; RRID: AB_2534006
IRDyeÒ 800CW donkey anti-rabbit	LI-COR	Cat# 925-32213; RRID: AB_2715510
Donkey anti-Sheep IgG (HL) Alexa Fluor™ 680	Invitrogen	Cat# A-21102; RRID: AB_2535755
Rabbit anti-Phospho-Histone H3 Ser10	Merck Millipore	Cat# 06-570; RRID: AB_310177
Sheep polyclonal Aurora A	Girdler et al., 2006^81^	N/A
Mouse monoclonal anti-PML (PG-M3)	Santa Cruz Technology	Cat# sc-966; RRID: AB_628162
Sheep polyclonal anti-TAO1	Westhorpe et al., 2010^82^	N/A
Rabbit monclonal anti-SMAD2 (D43B4)	Cell Signalling Technology	Cat# 5339; RRID: AB_10626777
Rabbit polyclonal anti-SNIP1	Proteintech	Cat# 14950-1-AP; RRID: AB_2286497
Goat polyclonal anti-UBE2I/UBC9	Abcam	Cat# ab21193; RRID: AB_2210477
Rabbit Recombinant anti-SAE2 [EPR14880]	Abcam	Cat# ab185955; RRID: AB_2827736
Rabbit monoclonal anti-cMYC [Y69]	Abcam	Cat#ab32072; RRID: AB_731658

**Bacterial and virus strains**		

XL1-Blue Competent Cells	Agilent Technologies	Cat# 200249

**Biological samples**		

Patient Samples	MCRC Biobank Manchester	N/A

**Chemicals, peptides, and recombinant proteins**		

(+)-Sodium I-ascorbate	Sigma Aldrich	Cat# A4034
17B Estradiol	Sigma Aldrich	Cat# E2758
2-Chloroacetamide (CAA)	Sigma Aldrich	Cat# 22790
alpha-tocopherol phosphate	Sigma Aldrich	Cat# T2020
Antipain	Sigma Aldrich	Cat# 10791
Aprotinin	Sigma Aldrich	Cat# A6279
Ascorbic acid	Sigma Aldrich	Cat# A5960
Bestatin	Sigma Aldrich	Cat# B8385
Blasticidin	Melford Scientific	Cat# B12150-0.1
Bovine Serum Albumin (BSA)	Sigma Aldrich	Cat# A2153
Bromophenol blue	Sigma Aldrich	Cat# B8026
Calcium Chloride	Thermo Fisher Scientific	Cat# C/1500/53
Cholera toxin	Sigma Aldrich	Cat# C8052
Cholesterol	Sigma Aldrich	Cat# C3045
Choline chloride	Sigma Aldrich	Cat# C7527
Chymostatin	Sigma Aldrich	Cat# 230790
COH000	Selleckchem	Cat# S0309
Crystal Violet	Sigma Aldrich	Cat# C0775
Dithiothreitol	Sigma Aldrich	Cat# D0632
EGF	Sigma Aldrich	Cat# E9644
Elacridar	Selleckchem	Cat# S7772
Ergocalciferol	Sigma Aldrich	Cat# E5750
Ethylenediaminetetraacetic acid (EDTA)	Thermo Fisher Scientific	Cat# D/0700/53
ethylene glycol-bis(β-aminoethyl ether)-N,N,N′,N′-tetraacetic acid (EGTA)	Sigma Aldrich	Cat# E4378
Folic acid	Sigma Aldrich	Cat# F8758
Formaldehyde solution (37% w/v)	Sigma Aldrich	Cat# F1635
Glycine	Fisher Scientific	Cat# BP381
Glycerol	Sigma Aldrich	Cat# G9012
HEPES	Sigma Aldrich	Cat# H4034
Hoechst 33258	Sigma Aldrich	Cat# B1155
Hydrocortisone	Sigma Aldrich	Cat# H0888
Hygromycin B	Merck Millipore	Cat# 10843555001
Hypoxanthine	Sigma Aldrich	Cat# H9636
i-Inositol	Sigma Aldrich	Cat# I75008
Insulin	Sigma Aldrich	Cat# I9278
L-Glutamine	Sigma Aldrich	Cat# 25030024
Leupeptin	Sigma Aldrich	Cat# L2884
Lipofectamine 2000	Thermo Fisher Scientific	Cat# 11668019
Lipoic acid	Sigma Aldrich	Cat# T1395
Magnesium chloride	Sigma Aldrich	Cat# M8266
Medium 199 (10x)	Life Technologies	Cat# 11825015
ML-792 (SAEi)	MedKoo Biosciences	Cat# 407886
Nutrient Mixture F12-Ham	Sigma Aldrich	Cat# N6760
OCMI (also available from USBiological #506390)	Ince et al., 2015^83^	N/A
O-phosphorylethanolamine	Sigma Aldrich	Cat# P0503
Penicillin-Streptomycin	Sigma Aldrich	Cat# 15140122
Pepstatin A	Sigma Aldrich	Cat# P5318
Phenylmethylsulfonyl fluoride	Sigma Aldrich	Cat# 93482
Polybrene	Sigma Aldrich	Cat# TR-1003-G
Propidium iodide	Sigma Aldrich	Cat# P4170
Retinoic Acid	Sigma Aldrich	Cat# R2625
Ribose	Sigma Aldrich	Cat# R9629
RNase A	Thermo Fisher Scientific	Cat# R1253
Selenious acid	Sigma Aldrich	Cat# 211176
Sodium chloride	Thermo Fisher Scientific	Cat# S/3160/65
Sodium deoxycholate	Sigma Aldrich	Cat# D6750
Sodium dodecyl sulphate	Sigma Aldrich	Cat# 75746
Sodium orthovanadate	Sigma Aldrich	Cat# S6508
Tetracycline	Sigma Aldrich	Cat# T7660
Thamine HCL	Sigma Aldrich	Cat# T1270
Transferrin	Sigma Aldrich	Cat# T8158
Tridothyronine	Sigma Aldrich	Cat# T2877
Triton X-100	Sigma Aldrich	Cat# T8787
Trypsin sequencing grade, modified	Sigma Aldrich	Cat# 11418025001
Tween-20	Sigma Aldrich	Cat# P2287
Uracil	Sigma Aldrich	Cat# U1128
Vitamin B12	Sigma Aldrich	Cat# V6629
Xanthine	Sigma Aldrich	Cat# X4002
ZM447439 (Aurora Bi)	AstraZeneca/Tocris	Cat# 2458

**Critical commercial assays**		

cobas® DNA Sample Preparation Kit	Roche	Cat#05985536190
TruSeq® DNA PCR-Free Kit	Illumina Inc	Cat#20015962
TruSeq® Stranded mRNA	Illumina Inc	Cat# 20020594
Stranded mRNA Prep Ligation kit	Illumina Inc	Cat# 20040532
RNeasy Plus Mini Kit	Qiagen	Cat# 74134
RNeasy Plus Micro Kit	Qiagen	Cat# 74004
SuperscriptTM III One-Step RT-PCR Platinum Taq HiFi	Thermo Fisher Scientific	Cat#12574035

**Deposited data**		

Code for analysis of RNA sequencing using MYC-resistant housekeeper genes (this study)	Zenodo	Zenodo DOI: https://doi.org/10.5281/zenodo.15125928
RNA sequencing of ovarian cancer models (27 published previously)	Nelson et al. 2020^52^; Barnes et al. 2021^48^; Coulson-Gilmer et al. 2021.^50^ EBML-EBI, European Nucleotide Archive	EMBL-EBI: E-MTAB-7223; EMBL-EBI: E-MTAB-10801; EMBL-EBI: E-MTAB-11000
RNA sequencing of ovarian cancer models new to this study	EBML-EBI, European Nucleotide Archive	EMBL-EBI: E-MTAB-14568
RNA sequencing of MYC-Low and MYC-High cells (this study)	EBML-EBI, European Nucleotide Archive	EMBL-EBI: E-MTAB-14654

**Experimental models: Cell lines**		

AAV293T	Agilent Technologies	Cat# 240073
CAOV3	ATCC	Cat# HTB-75; RRID: CVCL_0201
COV318	Sigma Aldrich	Cat# 07071903; RRID: CVCL_2419
COV362	Sigma Aldrich	Cat# 07071910; RRID: CVCL_2420
Kuramochi	JCRB Cell Bank	Cat# JCRB0098; RRID: CVCL_1345
NIHOVCAR3	ATCC	Cat# HTB-161; RRID: CVCL_0465
OV56	Sigma Aldrich	Cat# 96020759; RRID: CVCL_2673
OVISE	JCRB Cell Bank	Cat# JCRB1043; RRID: CVCL_3116
OVMANA	JCRB Cell Bank	Cat# JCRB1045; RRID: CVCL_3111
OVSAHO	JCRB Cell Bank	Cat# JCRB1046; RRID: CVCL_3114
RMG1	JCRB Cell Bank	Cat# JCRB0172; RRID: CVCL_1662
RKO Flp-In™ T-Rex™	Topham et al., 2015^39^	N/A
RKO Flp-In™ T-Rex™ FC-MYC	Littler et al., 2019^38^	N/A

**Oligonucleotides**		

siRNAs	Horizon Discovery	Tables S2 and S3
Primer: XhoI TP53 5′- CACCTCGAGGAGGA GCCGCAGTCAGATCCTA	Invitrogen	N/A
Primer: NotI TP53 3′- CACGCGGCCGCTCACAGTCTG AGTCAGGCCCTTCTGTC	Invitrogen	N/A
*TP53* sequencing primers: 5-CACCAGCAGCTCCTACACCG-3′ 5′-ATGAGCGCTGCTCAGATAGCG-3′	Invitrogen	N/A
*TP53* sequencing primers: 5-CGGCTCATAGGGCACCACC-3′ 5-TCTTCTTTGGCTGGGGAGAGG-3′	Invitrogen	N/A

**Recombinant DNA**		

pLVX-myc-GFP-H2B-Puro	Pillay et al., 2019^84^	N/A
psPAX2	Didier Trono unpublished	RRID: Addgene_12260
pMD2.G	Didier Trono unpublished	RRID: Addgene_12259
pBluescript SK-vector	Agilent Technologies	Cat#212206

**Software and algorithms**		

Adobe Photoshop® CC 2024	Adobe Systems Inc.	RRID:SCR_014199
Bestus Bioinformaticus Duk (BBDuk)	Bestus Bioinformaticus	RRID:SCR_016969; https://sourceforge.net/projects/bbmap/
bcl2fastq	Illumina Inc	RRID:SCR_015058; https://support.illumina.com/sequencing/sequencing_software/bcl2fastq-conversion-software.html
ChemiDoc™ Touch Imaging system	BioRad	Cat# 1708370
Columbus™ Image Data Storage and Analysis System	PerkinElmer	Cat# Columbus RRID:SCR_011436
CoolSNAP HQ^2^ camera	Photometrics	N/A
Excel	Microsoft	RRID:SCR_016137
FastQC	Babraham Bioinformatics	RRID:SCR_014583; https://www.bioinformatics.babraham.ac.uk/projects/fastqc/
FastQ Screen	Babraham Bioinformatics	RRID:SCR_000141; https://www.bioinformatics.babraham.ac.uk/projects/fastq_screen/
Flowjo software	FlowJo, LLC	RRID:SCR_008520
Harmony High Content Imaging and Analysis Software	PerkinElmer	Part# HH17000001; RRID:SCR_018809
Illustratorâ CC 2018	Adobe Systems Inc.	RRID: SCR_010279
ImageJ with the ColonyArea plugin	National Institute of Health	RRID: SCR_003070
Image Lab software	BioRad	
IncuCyteS3â Live Cell Analysis System	Sartorius AG	RRID:SCR_023147
IncuCyteS3â Cell-By-Cell Analysis Software Module	Sartorius AG	Cat# 9600-0031
MetaMorphÒ Microscopy Automation & Image Analysis Software	Molecular Devices	RRID:SCR_002368
OdysseyÒ CLx Imaging System	LI-COR	RRID:SCR_014579
Prism 10	GraphPad	RRID: SCR_002798
R package LIMMA	Bioconductor	RRID:SCR_010943; https://www.bioconductor.org/packages/release/bioc/html/limma.html
R package DESeq2	Bioconductor	RRID:SCR_015687; https://bioconductor.org/packages/release/bioc/html/DESeq2.html
R package gprofiler2	Raudvere et al.*,* 2019^85^	RRID:SCR_018190; https://biit.cs.ut.ee/gprofiler/page/r
R package mclust	CRAN	https://cran.r-project.org/web/packages/mclust/index.html
STAR	Dobin et al.*,* 2013^86^	RRID:SCR_004463

**Other**		

25 cm^2^ flasks	Corning	Cat# 430639
6-well plates	Corning	Cat# 353046
75 cm^2^ flasks	Corning	Cat# 430641
96-well black μclearâ plates	Greiner Bio-One	Cat# 655087
96-well CellCarrier plates	PerkinElmer	Cat# 6005550
Bambanker™	Wako pure chemical ind. Ltd	Cat# 302-14681
Bradford reagent	Sigma Aldrich	Cat# B6916
Cell+ 25 cm^2^ flasks	Sarstedt	Cat# 83.3910.300
Cell+ 75 cm^2^ flasks	Sarstedt	Cat# 83.3911.302
DharmaFECT 1	Horizon Discovery	Cat# T-2001-03
Dulbecco’s Modified Eagle Medium (DMEM)	Life Technologies	Cat# 41966052
EZ-Chemiluminescence Detection Kit for HRP	Geneflow Limited	Cat# KI-0172
Fetal Bovine Serum (Heat Inactivated)	Life Technologies	Cat# 10500064
H_2_O (molecular grade)	Merck Millipore	Cat# H2OMB0106
Immobilon-FL PVDF membrane	Merck Millipore	Cat# IPFL00010
Immobilon-P PVDF Membrane	Merck Millipore	Cat# IPVH00010
Luminata Forte Western HRP Substrate	Merck Millipore	Cat# WBLUF0100
NuPAGE™ 4-12% Bis-Tris protein gels (1.0mm)	Life Technologies	Cat# NP0321BOX
Opti-MEM™	Life Technologies	Cat# 11058021
Primaria™ 25 cm^2^ flasks	Corning	Cat# 353808
Primaria™ 75 cm^2^ flasks	Corning	Cat# 353810
Primaria™ 6 well plates	Corning	Cat# 353846
Primaria™ 24 well plates	Corning	Cat# 353847
QIAprep Spin Miniprep Kit	Qiagen	Cat# 27104
Qubit 4 Fluorometer	Thermo Fisher Scientific	Cat# Q33238
REVERT solution	LI-COR	Cat# 926-11015
RPMI 1640 Medium	Life Technologies	Cat# 21875034

### Experimental model and study participant details

#### Patient sample collection

Thirty research samples from 25 patients (age range 25–84 years) were obtained with informed patient consent from the Manchester Cancer Research Centre (MCRC) Biobank, which is licensed by the Human Tissue Authority (license number: 30004) and ethically approved as a research tissue bank by the South Manchester Research Ethics Committee (Ref: 22/NW/0237). Note that ancestry, race, and ethnicity are not routinely collected by the MCRC Biobank. The role of the MCRC Biobank is to distribute samples and does not endorse studies performed or the interpretation of results. For more information, see https://www.mcrc.manchester.ac.uk/research/mcrc-biobank.

#### *Ex vivo* ovarian cancer models

Thirty OCMs were generated from ascitic fluid samples, 28 of which are published previously (Table S1).^48^^,^^50^^,^^51^^,^^52^^,^^87^ To establish OCMs first described here,^52^ ascites were centrifuged, red blood cells removed, then remaining cells plated into OCMI media^83^ in Primaria or Cell+ flasks and incubated at 37°C for 2–4 days in a humidified 5% CO_2_ and 5% O_2_ atmosphere. Media was then replaced every 3–4 days and, once attached, selective trypsinisation used to separate stromal from tumour cells. Established OCMs were cultured in OCMI.^52^^,^^83^ For long-term storage, OCMs were frozen in Bambanker and stored in LN_2_.

#### Cell lines

RKO Flp-In™ T-Rex™ cells^39^ and AAV293T (Agilent Technologies) were cultured in Dulbecco’s Modified Eagle Medium (DMEM). RKO Flp-In™ T-Rex™ FC7-MYC cells were cultured in DMEM with 100 ng/ml tetracycline, 8 μg/ml blasticidin and 400 μg/ml hygromycin B (Sigma Aldrich), as described previously.^38^ The human ovarian carcinoma cell lines OVCAR3 (ATCC, RRID: CVCL_0465), Kuramochi (RRID: CVCL_1345), OVMANA (RRID: CVCL_3111), OVSAHO (RRID: CVCL_3114) and OVISE (RRID: CVCL_3116), all JCRB Cell Bank, were cultured in RPMI 1640 medium; COV362 (RRID: CVCL_2420), COV318 (RRID: CVCL_2419), both Sigma Aldrich, and CAOV3 (ATCC, RRID: CVCL_0201) in DMEM; RMG1 (RRID: CVCL_1662, JCRB Cell Bank) in Ham’s F12 medium (Sigma Aldrich). All of the above cell lines were grown with 10% Fetal Bovine Serum (FBS), 100 U/ml penicillin, 100 μg/ml streptomycin and 2 mM glutamine and maintained at 37°C in a humidified 5% CO_2_ atmosphere. OV56 (RRID: CVCL_2673, Sigma Aldrich) was grown in DMEM/F12 with 5% FBS, 10 μg/ml insulin, 0.5 μg/ml hydrocortisone, 100 μg/ml streptomycin, 100 U/ml penicillin, 2 mM glutamine and maintained at 37°C in a humidified 5% CO_2_ atmosphere. Cells were periodically tested for the presence of mycoplasma and authenticated (Promega Powerplex 21 System) by the Molecular Biology Core Facility at the CRUK Manchester Institute.

### Method details

#### DNA sequencing

##### TP53 genotyping of primary tumours

FFPE tumour blocks retrieved by the MCRC Biobank were genotyped by Manchester Centre for Genomic Medicine, St Mary’s Hospital (Table S1). As described previously,^52^ blocks were assessed for total cellularity and neoplastic cell content (percentage of all nucleated cells on a Haematoxylin and Eosin-stained slide), with neoplastic cell count ≥10% required. Tumour from 5 × 5 μM unstained pathology slides underwent DNA extraction using the cobas® DNA Sample Preparation Kit (Roche), and DNA was quantified using a Qubit 2.0 Fluorometer (ThermoScientific). After targeted enrichment (GeneRead Clinically Relevant Tumour Targeted Panel V2; Qiagen) and library preparation (TruSeq® DNA PCR-Free Kit; Illumina), sequencing was performed on an Illumina MiSeq platform using 2 x 150 paired-end sequencing chemistry. Target read depth across all coding regions (exon 2 to 9) was 350× minimum for somatic variants. Mutation names are according to Human Genome Variation Society guidelines (http://www.hgvs.org/; reference sequence NM_000546.5), with variant calls independently reviewed using a genome browser and BAM files (Integrated Genomic Viewer). At a variant allele frequency ≥4%, the call sensitivity was >90% and specificity >95% after manual review.

##### OCM TP53 genotyping

RNA was extracted using RNeasy Plus Mini kit (Qiagen) and *TP53* complementary DNA generated using Superscript™ III One-Step RT-PCR Platinum Taq HiFi (Thermofisher) and primers 5′-CACCTCGAGGAGGAGCCGCAGTCAGATCCTA; 3′-CACGCGGCCGCTCACAGTCTGAGTCAGGCCCTTCTGTC. PCR products cloned into pBluescript SK-vector were transformed into XL1-Blue competent cells, plasmid DNA extracted using QIAprep Spin Miniprep Kit (Qiagen) and sequenced using primers: 5′-CACCAGCAGCTCCTACACCG-3′, 5′-ATGAGCGCTGCTCAGATAGCG-3′, 5′-CGGCTCATAGGGCACCACC-3′, 5′-TCTTCTTTGGCTGGGGAGAGG-3′. Sequences were aligned using Seqman Pro (DNASTAR). For OCMs first described here the pBluescript-p53 vectors were subjected to whole-plasmid sequencing by nanopore (Plasmidsaurus).

#### Small-molecule inhibitors

All inhibitors were dissolved in DMSO. ML-792 (MedKoo Biosciences; SAEi), verified using 1H and 13C NMR, was used at a final concentration of 25 nM for RKO Flp-In™ T-Rex™ and RKO Flp-In™ T-Rex™ FC-MYC cells, 200 nM for ovarian cancer cell lines, and 100 nM and 500 nM for OCMs. A titration of 15 nM to 4 μM ML-792 was used for screening the ovarian cancer cell lines and OCMs. Other inhibitors were used at final concentrations: elacridar, 250 nM; ZM447439 (Aurora Bi), 1 μM.

#### Generation of cells stably expressing GFP-tagged histone H2B

AAV293T cells were plated at 5×10^4^ cells/well in a 24-well plate. Media was replenished 1 h before transfection. Cells transfected with pLVX-myc-EmGFP-H2B^84^ plus psPAX2 and pMD2.G (gifts from Didier Trono; Addgene plasmid #12260; http://n2t.net/addgene:12260; RRID: Addgene_12260 and Addgene plasmid #12259; http://n2t.net/addgene:12259; RRID: Addgene_12259), using 16.6 mM CaCl_2_ in DMEM supplemented with 10% Hyclone™ serum (GE Healthcare), were incubated overnight. Virus was harvested 48 h after transfection, centrifuged and filtered (0.45 μm). OCMs were seeded at 2×10^5^ cells/well in a 12-well plate, and diluted lentivirus with 10 μg ml/ml polybrene added 48 h later. Plates were centrifuged at 300×g at 30°C for 2.5 h. One millilitre of media was added and plates incubated overnight. Puromycin (1 μg/ml) was added 48 h post-transduction.

#### Drug-sensitivity, proliferation, and cell fate profiling using high-throughput timelapse microscopy

RKO cells and ovarian cancer cell lines were seeded in black μclear® 96-well plates (Greiner Bio-One) at 1000–19000 cells/well 24 h prior to drug addition. Plates were maintained at 37°C in a humidified 5% CO_2_ atmosphere and imaged using an IncuCyte® S3 (Sartorius AG) with a 20x objective. Nine phase contrast images/well were collected every two hours when analysing proliferation and drug sensitivity, or every 10 min for cell fate profiling. IncuCyte® S3 phase cell-by-cell module was used in real time to measure phase object count for label-free counting. For cell fate profiling, image sequences were exported to MPEG-4 and manually annotated with cell behaviours.^88^ Timing data were imported into Prism 10 (GraphPad) for statistical analysis and presentation.

OCMs expressing a GFP-tagged Histone were seeded in black μclear® 96-well plates (Greiner Bio-One) at 1000–12000 cells/well 24 h prior to drug addition. Plates were imaged as above, with phase and fluorescent images collected. IncuCyte® S3 software was used in real time to measure green object count as a proxy for proliferation, and cell fate profiling was conducted as above without fluorescence. For drug sensitivity, the Area Under the Curve (AUC) at each drug concentration was calculated and plotted against drug concentration to generate dose-response curves from which GI_50_ values were calculated.

#### Colony formation assay

Cells were seeded into 6-well plates at 500 cells/well for ovarian cancer cell lines, or 1000–2000 cells/well for OCMs, 24 h prior to drug addition. Media containing drugs was replaced every 3–4 days, with fixation of cells in 1% (v/v) formaldehyde (Fisher Scientific) occurring when colonies had developed. Cells were stained with 0.05% (w/v) crystal violet (Sigma-Aldrich), before imaging using a ChemiDoc™ Touch Imaging System (BioRad). Colony area quantification used ImageJ software (NIH) and the ColonyArea plugin.

#### Immunoblotting

Cell pellets were boiled in SDS sample buffer (0.35 M Tris pH 6.8, 0.1 g/ml sodium dodecyl sulphate, 93 mg/ml dithiothreitol, 30% (v/v) glycerol, 50 μg/ml bromophenol blue; all Sigma Aldrich) and proteins resolved by SDS-PAGE using NuPAGE™ 4–12% (v/v) Bis-Tris protein gels (1.0 mm) (Life Technologies), before electroblotting onto methanol-soaked Immobilon-P PVDF membranes (Merck Millipore). Membranes were blocked in 5% (w/v) dried skimmed milk (Marvel) dissolved in TBS-T, (50 mM Tris pH 7.6, 150 mM NaCl, 0.1% Tween-20) and incubated at 4°C overnight with primary antibodies (see key resources table – c-MYC 1:3000; TAO1 1:1000^82^; UBC9 1:1000; SNIP1 1:1000; SMAD2 1:1000). Membranes were washed three times TBS-T before incubating with horseradish-peroxide (HRP)-conjugated secondary antibodies (key resources table) for 2 hours. After three TBS-T washes, bound secondary antibodies were detected using either EZ-Chemiluminescence Reagent (Geneflow Ltd) or Luminata™ Forte Western HRP Substrate (Merck Millipore) and a ChemiDoc™ Touch Imaging System (BioRad). Image Lab software (BioRad) and Adobe Photoshop® CC 2024 (Adobe Systems Inc.) were used for image processing.

LI-COR immunoblotting was performed as previously described.^50^ In brief, proteins were extracted, quantified by Bradford, and boiled in SDS sample buffer, before SDS-PAGE and electroblotting onto Immobilon-FL PVDF membrane (Millipore; LI-COR). REVERT total protein stain solution was imaged for loading normalisation, before using REVERT reversal solution (0.1 M NAOH, 30% v/v methanol in water) and blocking in either 5% (w/v) dried skimmed milk (Marvel) or 5% (w/v) bovine serum albumin (BSA, Sigma Aldrich) dissolved in TBS-T for phospho-specific antibodies. Primary antibody incubation was as described above (c-MYC 1:3000; SAE2 1:800). Membranes were then incubated with fluorescently conjugated secondary antibodies (key resources table) diluted 1:10000 in 5% dried skimmed milk in 0.2% Tween-20, 0.01% SDS TBS for 1 hour, then rinsed with TBS. Membranes were imaged using Odyssey® CLx Imaging System (LI-COR). Relative quantity values were calculated using Image Studio™ software.

#### Immunofluorescence

Cells were seeded 24 h prior to drug treatment onto collagen-coated 19 mm coverslips at 8 × 10^4^ (RKO) or 3 × 10^4^ cells (OCM.72) per coverslip or 96-well Cell Carrier plates (PerkinElmer) at 2000 cells/well (OCM.72). Cells were fixed with 3.7% (v/v) formaldehyde for 15 min, washed in PBS, before quenching in glycine (12.5 mM in PBS) for 5 min. Subsequent washes used PBS-T (PBS, 0.1% (v/v) Triton X-100). Cells were incubated with primary antibodies in PBS-T (see key resources table – Aurora A 1:1000^81^; pH3 1:1000; PML 1:100) for 30 min at room temperature before washing and incubation with fluorescently conjugated secondary antibodies (1:500) for 30 min at room temperature (see key resources table). Cells were washed, DNA stained with 1 μg/ml Hoechst 33258 for 1 min at room temperature, followed by further washes. Coverslips were mounted onto microscope slides (90% glycerol, 20 mM Tris, pH 9.2) and 40x objective was used on an Axioskop2 (Carl Zeiss, Inc) microscope fitted with a CoolSNAP HQ camera (Photometrics) for image acquisition. Images were processed using MetaMorph Software (Molecular Devices) with Adobe Photoshop® CC 2024 (Adobe Systems Inc.). For high-throughput immunofluorescence, an Operetta® High Content Imaging System (Perkin Elmer) was used with Harmony and Columbus High Content Imaging and Analysis Software (Perkin Elmer), with Hoechst 33258 staining used to calculate intensity thresholds and generate a nuclear mask. For PML foci analysis, foci size and number were quantitated from within the nuclear area and used to calculate large PML foci percentage.

#### RNA interference

Reverse transfection used Opti-MEM media, DharmaFECT-1 transfection reagent (Dharmacon/Horizon Discovery) and siRNA at a final concentration of 66 nM, according to manufacturer’s instructions; all SMARTpools consisting of four individual ON-TARGETplus or siGENOME (E3 SUMO ligase screen) oligonucleotides from Horizon Discovery (Tables S2 and S3).

For flow cytometry, immunoblotting and high-throughput time-lapse microscopy of RKO Flp-In™ T-Rex™ FC-MYC cells, cells were mixed with transfection reagent and seeded at 3 × 10^5^ cells/well of a 6-well plate for flow cytometry, 1.7 × 10^5^ cells/ml of a 24-well plate for immunoblotting, or 0.8 × 10^5^ cells/ml of a 96-well plate for high-throughput time-lapse microscopy, in the presence or absence of 500 ng/ml tetracycline. Cells were incubated for 72 or 96 hours, before either being fixed and stained according to the flow cytometry protocol, harvested for immunoblotting, or imaged as outlined for proliferation by high-throughput time-lapse microscopy, AUC values determined, and data visualised using GraphPad Prism 10.

For high-throughput time-lapse microscopy of OCMs, cells were mixed with transfection reagents and seeded at 2000–4000 per well into collagen-coated black μclear® 96-well (Greiner BioOne). Time courses are described in the figure legends. OCM cells were imaged as outlined for proliferation by high-throughput time-lapse microscopy for 6 days, AUC values determined, and data visualised using GraphPad Prism 10.

#### Flow cytometry

For DNA content analysis, RKO cells, ovarian cancer cell lines and OCM.72 were exposed to drugs for 72, 48 or 96 hours, respectively, harvested, washed in PBS, fixed in ice-cold 100% ethanol and stored at −20°C overnight. Cells were washed twice in PBS and stained with propidium iodide (40 μg/mL) (Sigma) with RNase A (50 μg/ml) (Thermo Scientific) for 30 min at room temperature. Cells were analysed using an Attune™ NxT flow cytometer (ThermoFisher Scientific) or stored at 4°C prior to analysis. Data were analysed using FlowJo software (FlowJo, LLC).

#### High-resolution timelapse microscopy

High-resolution time-lapse microscopy utilised an Axiovert 200 manual microscope (Carl Zeiss, Inc.) with an automated stage (Nano-Drive™C; Mad City Labs, Inc.) and environmental control chamber (Solent Scientific) to maintain cells at 37°C in a humidified stream of 5% CO_2_. A 40× Plan-NEOFLUAR objective was used for imaging. Shutters, filter wheels and point visiting were driven by MetaMorph software (Molecular Devices) and images taken using an Evolve delta camera (Photometrics). Image sequences were exported and analysed manually.

#### RNA sequencing

RKO Flp-In™ T-Rex™ FC-MYC cells were plated into T75 flasks at 3 × 10^5^ (MYC-high) or 4 × 10^5^ (MYC-low) cells/flask with either DMSO or 25 nM ML-792 ± 500 ng/mL tetracycline. After 24 h, cells were scraped into the media, centrifuged and resuspended in PBS, pelleted and stored at −80°C before RNA extraction using RNeasy Micro Kit (Qiagen) following manufacturer’s instructions. RNA was quantified using a NanoDrop One (Thermo Scientific) and 1 μg stored at −80°C before RNA-seq by the Genomic Technologies Core Facility. Libraries were generated using the TruSeq® Stranded mRNA assay (Illumina, Inc.) following manufacturer’s instructions. Adaptor indices were used to allow multiplexing of pooled libraries before cluster generation using a cBot instrument (Illumina, Inc.). The final library was paired-end sequenced (76 + 76 cycles, plus indices) on an Illumina HiSeq4000 instrument.

RNA-seq of OCMs was as largely as previously described.^48^^,^^50^^,^^52^ Total RNA extracted using a RNeasy Plus Mini kit (Qiagen) was submitted to the Genomic Technologies Core Facility. After assessment for quality and integrity using a 4200 TapeStation (Agilent Technologies), libraries were generated using the Illumina® Stranded mRNA Prep Ligation kit (Illumina, Inc.) according to the manufacturer’s instructions. Briefly, polyadenylated mRNA was purified from total RNA (typically 0.025–1 μg) using poly-T oligo-attached magnetic beads. mRNA was fragmented under elevated temperature before being reverse transcribed into first-strand cDNA using random hexamer primers in the presence of Actinomycin D. Following removal of template RNA, second-strand cDNA was synthesized to yield blunt-ended, double-stranded cDNA fragments (strand specificity maintained by incorporation of dUTP in place of dTTP to quench the second strand during subsequent amplification). Following a single adenine base addition, adapters with a complementary thymine overhang were ligated to the cDNA fragments followed by ligation of pre-index anchors to prepare for dual indexing. PCR was then used to add index adapter sequences to enable multiplexing of the final cDNA libraries, which were pooled prior to loading on to an SP flow cell and paired-end sequenced (59 + 59 cycles, plus indices) on an Illumina NovaSeq6000 instrument.

Output data was demultiplexed and converted into FASTQ files using bcl2fastq software (Illumina, Inc., v2.20.0.422). Unmapped paired-end reads were quality assessed using FastQC (v0.11.3)^89^ and FastQScreen (RKO cells v0.13.0; OCMs v0.14.0),^90^ followed by adapter and low-quality base trimming with BBDuk (BBTools suite; RKOs v38.90; OCMs v36.32).^91^ Trimmed reads were mapped against the human reference genome (GRCh38) and gene annotation from Gencode (RKOs v37; OCMs v32) using STAR (RKOs v2.7.8a; OCMs v2.7.2b).^86^^,^^92^ The “–quantMode GeneCounts” option was used to obtain read counts per gene from STAR.

#### Differential gene expression analyses

R version 4.4.1 was used for all analyses. Differential gene expression analysis was performed using DESeq2 (v1.44.0)^93^ from Bioconductor (v3.19). *p* values were adjusted using the Benjamini Hochberg method, and those with an adjusted value < 0.05 were considered significant. Pathway analysis was performed using R package gprofiler2.^85^ gprofiler2 interfaces with Gene Ontology, KEGG, Reactome, regulatory motif matches from TRANSFAC, and protein complexes from CORUM. Genes upregulated and downregulated were handled separately.

##### Normalisation to MYC-resistant house-keeper genes

MYC unresponsive house-keeping genes were identified independently.^67^ Reads Per Kilobase per Million mapped reads (RPKM) normalised based on synthetic spike-ins were downloaded from GEO Series GSE40784. Lowly expressed genes were removed (RPKM <1), the coefficient of variation for each gene calculated, and genes with the lowest 2% variance retained as housekeeping genes non-responsive to MYC overexpression, identifying 219 genes. The list was filtered based on our own RNA-seq, retaining only genes with a total read count >3 (166 genes). These genes were used as a set of control genes for normalisation in DESeq2, instead of the usual size factor estimation method. Two DESeq2 models were created, one with a grouping variable to allow identification of genes that are differentially expressed between each pairwise condition and another that identifies the interaction term. The latter specifically is identifying genes that behave differently upon exposure to ML-792, depending on MYC level. Genes identified by the interaction analysis were clustered using K-means.

##### Primary screen of OCMs

Colony formation area and GI_50_ values were used to perform mixture modelling using the R package mclust (v6.1.1).^94^ Optimum clustering identified three clusters corresponding to OCMs with sensitive, intermediate and resistant response to ML-792. The sensitive and resistant clusters were used as discrete labels to input into DEseq2 to identify differentially expressed genes.

##### Second screen of OCMs

OCMs identified as elacridar-responders and non-responders were input into DESeq2 as a discrete variable. CFA at 100 nM and 500 nM ML-792 were centred and scaled and input into DESeq2 as continuous variables, identifying genes whose expression correlates with colony formation area.

#### Analysis of ectopic/endogenous *MYC* mRNA

As the ectopically introduced MYC transgene underwent site-directed mutagenesis of three individual nucleotides to prevent targeting by the sgRNA,^38^ this allowed quantification of *MYC* mRNA from the ectopic and endogenous locus. The three sites are 127,738,271 (C to G), 127,738,277 (T to C) and 127,738,283 (C to T) on chromosome 8 (human reference genome GRCh38). The number of wild-type and mutation-containing reads spanning these loci was quantified, averaged, and normalised to the total library size.

#### MYC target gene enrichment analysis

MYC target gene enrichment was tested using the ‘cameraPR’ function from the *limma* R package (v3.60.4),^95^ which accounts for inter-gene correlation between the input gene set.

#### Cancer cell line encyclopaedia (CCLE) analyses

The genetic background of the cell lines^47^ was visualised using the cBioPortal for Cancer Genomics (https://www.cbioportal.org/).^45^^,^^46^ Gene counts were downloaded from the Depmap data portal, release 24Q2. Hallmark MYC targets were downloaded from https://www.gsea-msigdb.org/gsea/msigdb/cards/HALLMARK_MYC_TARGETS_V1. Normalised gene expression for MYC target genes were z-scored and summed to give a single value for MYC target gene expression.

### Quantification and statistical analysis

Microsoft Excel was used for data organisation and Prism 10 (GraphPad) for statistical analysis, where ∗*p* < 0.05, ∗∗*p* < 0.01, ∗∗∗*p* < 0.001, ∗∗∗∗*p* < 0.0001, ns: *p* > 0.05. Details of statistical analyses are described in the figure legends.

## Supplemental information

Document S1. Figures S1–S7 and Table S1

Table S2. E3 SUMO ligases Human siRNA sequences Excel file related to Figure S2.

Table S3. ON-TARGETplus SMARTpool Human siRNA sequences Excel file related to Figures 6 and S6.
