# Supplementary material for: Targeting SUMOylation in ovarian cancer: Sensitivity, resistance, and the role of MYC
Source: iScience. 2025 Apr 29;28(6):112555. doi: 10.1016/j.isci.2025.112555 (PMC12145850; doi:10.1016/j.isci.2025.112555)
Supplement: Document S1. Figures S1–S7 and Table S1 [file mmc1.pdf]

iScience, Volume *28*

Supplemental information

Targeting SUMOylation in ovarian cancer: Sensitivity, resistance, and the role of MYC

Samantha Littler, Bethany M. Barnes, Rhys Owen, Louisa Nelson, Anthony Tighe, I-Hsuan Lin, Hugh C. Osborne, Christine K. Schmidt, Joanne C. McGrail, and Stephen S. Taylor

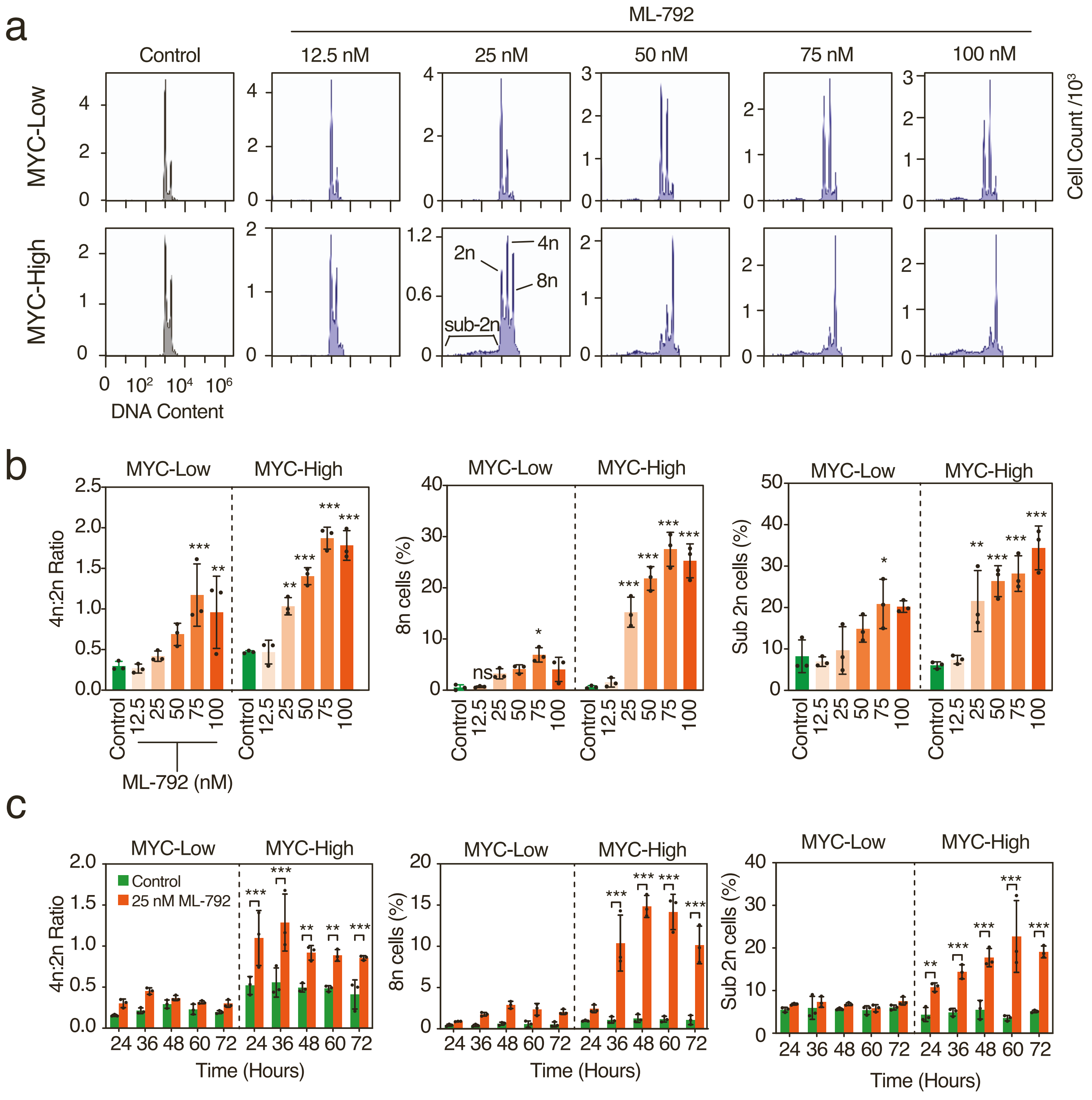
a
ML-792
Control
12.5 nM
25 nM
50 nM
75 nM
100 nM
MYC-Low
MYC-High
Cell Count /10³
2n
4n
8n
sub-2n
DNA Content
b
MYC-Low
MYC-High
4n:2n Ratio
8n cells (%)
Sub 2n cells (%)
Control
12.5
25
50
75
100
ML-792 (nM)
ns
c
Control
25 nM ML-792
4n:2n Ratio
8n cells (%)
Sub 2n cells (%)
Time (Hours)

- Figure S1 -

Figure S1. Pharmacological inhibition of SUMO signalling induces cell division failures in MYC-High cells. (**a**) DNA content histograms of FC-MYC cells exposed ML-792 at the concentrations indicated for 48 hours in the presence (MYC-High) or absence (MYC-Low) of 500 ng/mL tetracycline. (**b**) Bar graphs quantitating DNA content analysis showing the 4n:2n ratio, percentage of cells with 8n and sub 2n DNA contents. Data are mean ± SD from three biological replicates. Two-way ANOVA with Dunnett's multiple comparisons. (**c**) Quantification of DNA content analysis showing the 4n:2n ratio and percentages of cells with 8n and sub-2n DNA content for MYC-Low and MYC-High cells treated with 25 nM ML-792 for 24–72 hours. Data are mean ± SD from three biological replicates. Three-way ANOVA with Tukey's multiple comparisons. *$p<0.05$; **$p<0.01$, ***$p<0.001$, ns: $p>0.05$. Related to **Figure 1**.

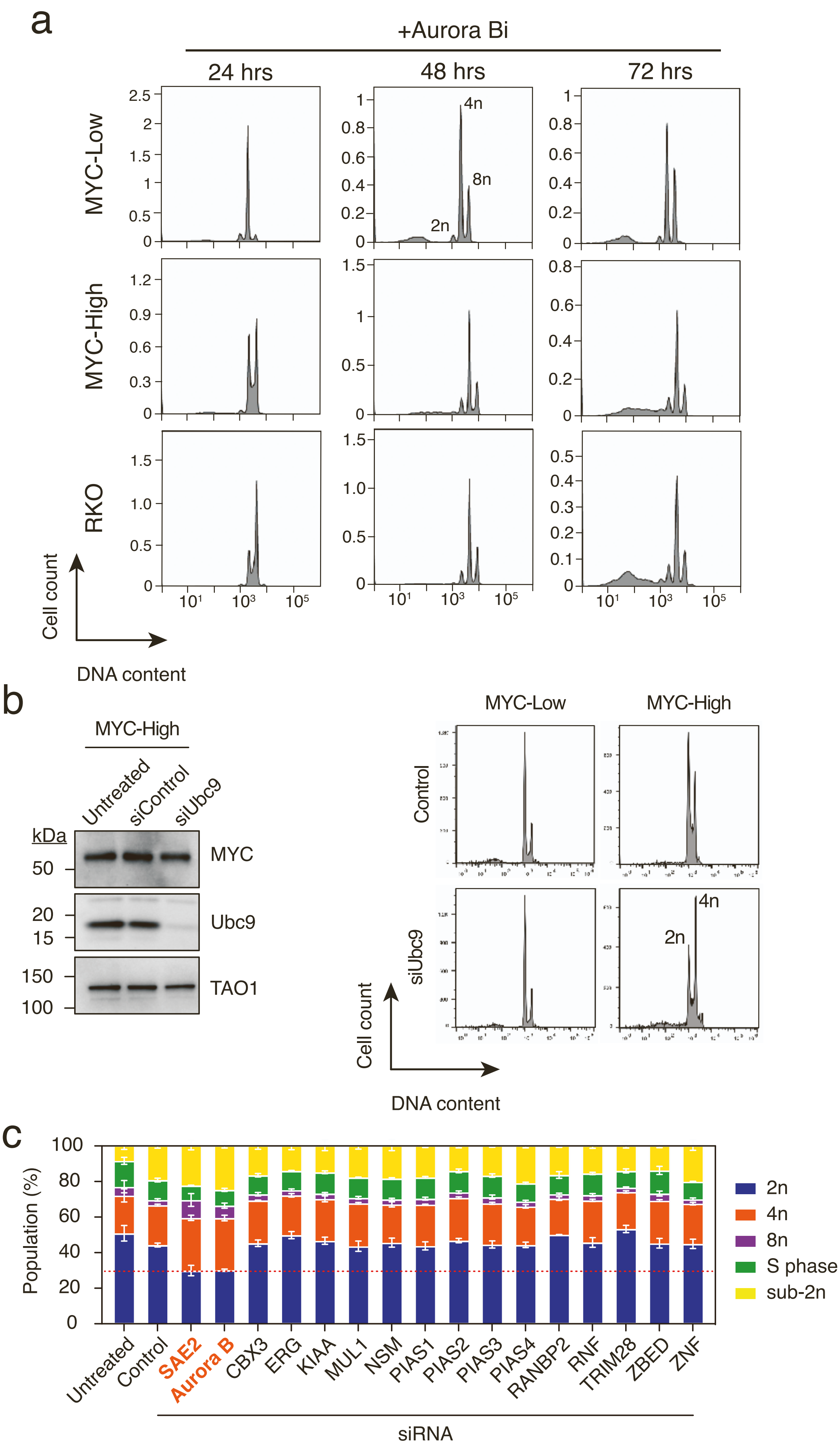
a
+Aurora Bi
24 hrs
48 hrs
72 hrs
MYC-Low
MYC-High
RKO
4n
8n
2n
Cell count
DNA content
b
MYC-High
Untreated
siControl
siUbc9
kDa
50
20
15
150
100
MYC
Ubc9
TAO1
MYC-Low
MYC-High
Control
siUbc9
4n
2n
Cell count
DNA content
c
Population (%)
Untreated
Control
SAE2
Aurora B
CBX3
ERG
KIAA
MUL1
NSM
PIAS1
PIAS2
PIAS3
PIAS4
RANBP2
RNF
TRIM28
ZBED
ZNF
siRNA
2n
4n
8n
S phase
sub-2n

- Figure S2 -

Figure S2. Inhibition of SUMO signalling induces cell division failures in MYC-High cells. (**a**) DNA content histograms of FC-MYC cells ± tetracycline and parental RKO cells exposed to 1 μM Aurora B inhibitor (AuroraBi) for 24, 48 and 72 hours. (**b**) Immunoblot and DNA content histograms following transfection of siRNAs targeting Ubc9 for 72 hours. TAO1 is used as a loading control. (**c**) Quantitation of DNA content analysis of MYC-High cells transfected with siRNAs targeting 14 SUMO E3 ligases, with SAE2 and Aurora B, and non-targeting siRNAs, as positive and negative controls, respectively. Data are mean ± SD from three biological replicates. Related to **Figure 1** and **Table S2**.

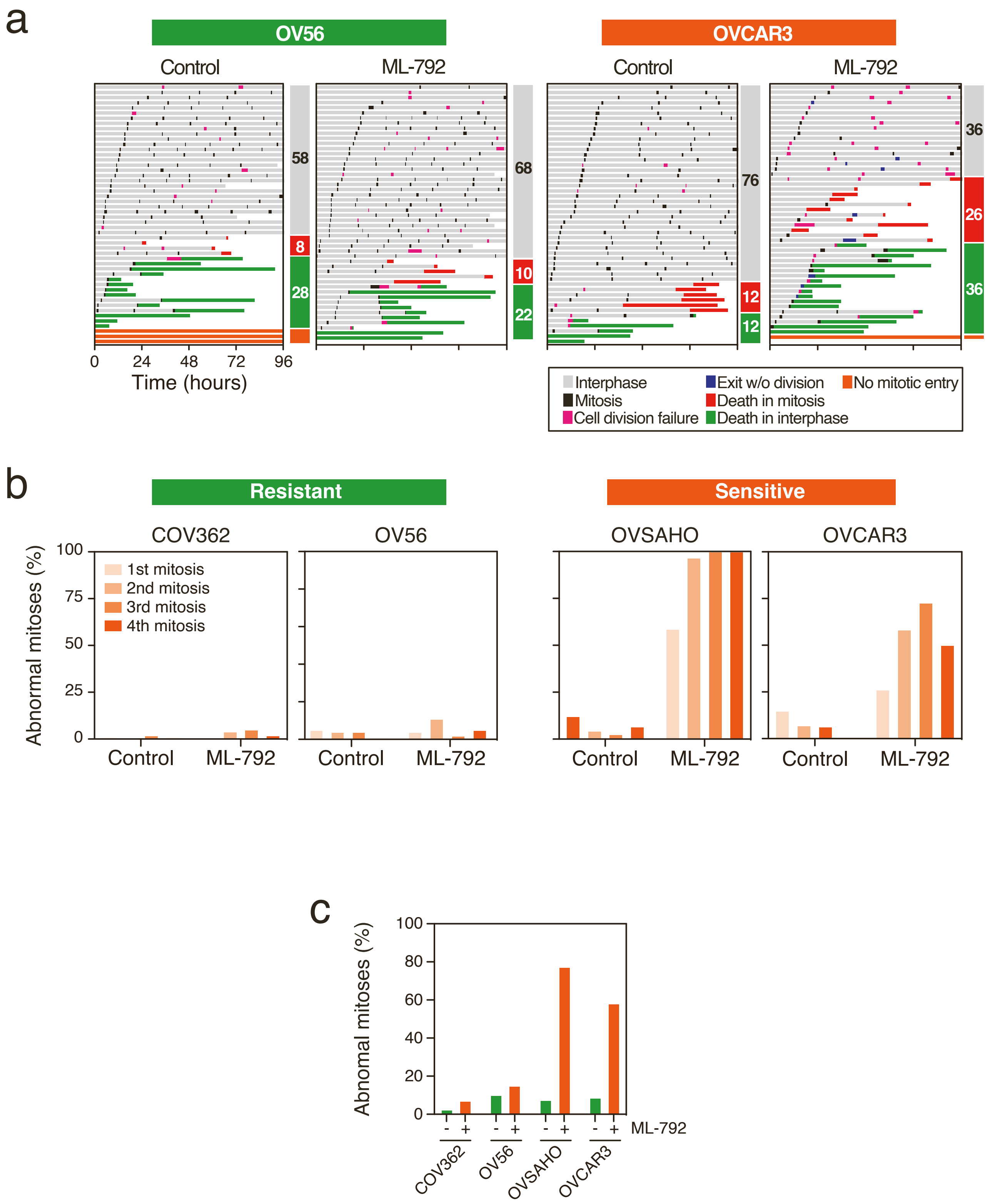
a
OV56
OVCAR3
Control
ML-792
Control
ML-792
58
8
28
68
10
22
76
12
12
36
26
36
0
24
48
72
96
Time (hours)
Interphase
Mitosis
Cell division failure
Exit w/o division
Death in mitosis
Death in interphase
No mitotic entry
b
Resistant
Sensitive
COV362
OV56
OVSAHO
OVCAR3
Abnormal mitoses (%)
100
75
50
25
0
1st mitosis
2nd mitosis
3rd mitosis
4th mitosis
Control
ML-792
c
Abnormal mitoses (%)
100
80
60
40
20
0
- +
ML-792
COV362
OV56
OVSAHO
OVCAR3

- Figure S3 -

Figure S3. A subset of ovarian cancer cell lines are sensitive to inhibition of SUMO signalling. (**a**) Cell fate profiles of ovarian cancer cell lines indicated exposed to 200 nM ML-792 for 96 hours. Horizontal bars represent single cells (50 cells per condition), with colours indicating cell behaviour. Numbers in coloured boxes show the percentage of cells with the indicated behaviour. (**b**) Bar graphs quantitating the percentage of cells that underwent an abnormal division during the 1st, 2nd, 3rd and 4th mitosis and (**c**) total percentage of abnormal mitoses. Related to **Figure 2**.

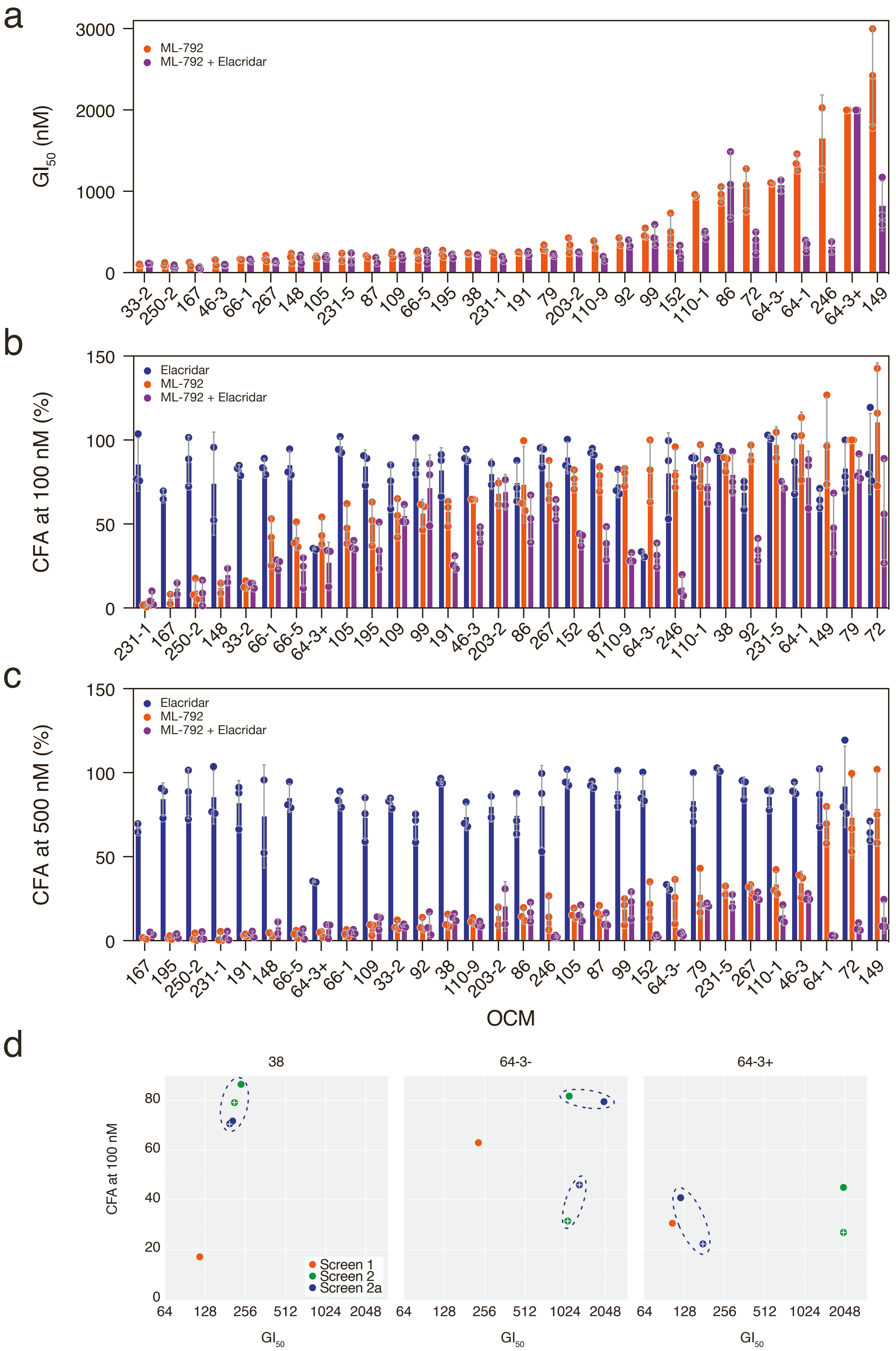
a
ML-792
ML-792 + Elacridar
GI50 (nM)
3000
2000
1000
0
33-2 250-2 167 46-3 66-1 267 148 105 231-5 87 109 66-5 195 38 231-1 191 79 203-2 110-9 92 99 152 110-1 86 72 64-3- 64-1 246 64-3+ 149
b
Elacridar
ML-792
ML-792 + Elacridar
CFA at 100 nM (%)
150
100
50
0
231-1 167 250-2 148 33-2 66-1 66-5 64-3+ 105 195 109 99 191 46-3 203-2 86 267 152 87 110-9 64-3- 246 110-1 38 92 231-5 64-1 149 79 72
c
Elacridar
ML-792
ML-792 + Elacridar
CFA at 500 nM (%)
150
100
50
0
167 195 250-2 231-1 191 148 66-5 64-3+ 66-1 109 33-2 92 38 110-9 203-2 86 246 105 87 99 152 64-3- 79 231-5 267 110-1 46-3 64-1 72 149
OCM
d
38
64-3-
64-3+
CFA at 100 nM
80
60
40
20
0
Screen 1
Screen 2
Screen 2a
64 128 256 512 1024 2048
GI50

- Figure S4 -

Figure S4. Screen 2: inhibition of drug efflux activity to redefine ML-792 sensitivity. (**a**) Bar graph plotting GI_{50} values derived from 144-hour GFP-H2B-based proliferation assays over a range of ML-792 concentrations ± 250 nM elacridar and ranked based on ML-792 sensitivity. (**b-c**) Quantification of colony formation area (CFA) as a percentage of untreated controls, for OCMs continuously treated with either 250 nM elacridar or 100 nM ML-792 (**b**) or 500 nM ML-792 (**c**) ± 250 nM elacridar and ranked based on ML-792 sensitivity. Data are mean ± SD from three biological replicates. (**d**) *xy* plot comparing mean GI_{50} values versus CFA at 100 nM ML-792 of three OCMs re-screened (Screen 2a). Symbol colour indicates screen; "+" represents the addition of elacridar; and dotted circles highlight the screen data included in further analyses. Note that in screen 1, OCM.38 was classified as sensitive; however, screen 2 and the rescreen indicate that it is more likely resistant. While OCM.64-3− was scored as intermediate in screen 1, it appeared relatively resistant in screen 2. The rescreen confirmed this and shows that it is an elacridar responder. OCM.64-3+ was initially categorised as sensitive but displayed a high GI_{50} value in screen 2. The rescreen reflected screen 1, indicating that it is indeed relatively sensitive. Related to **Figure 5**.

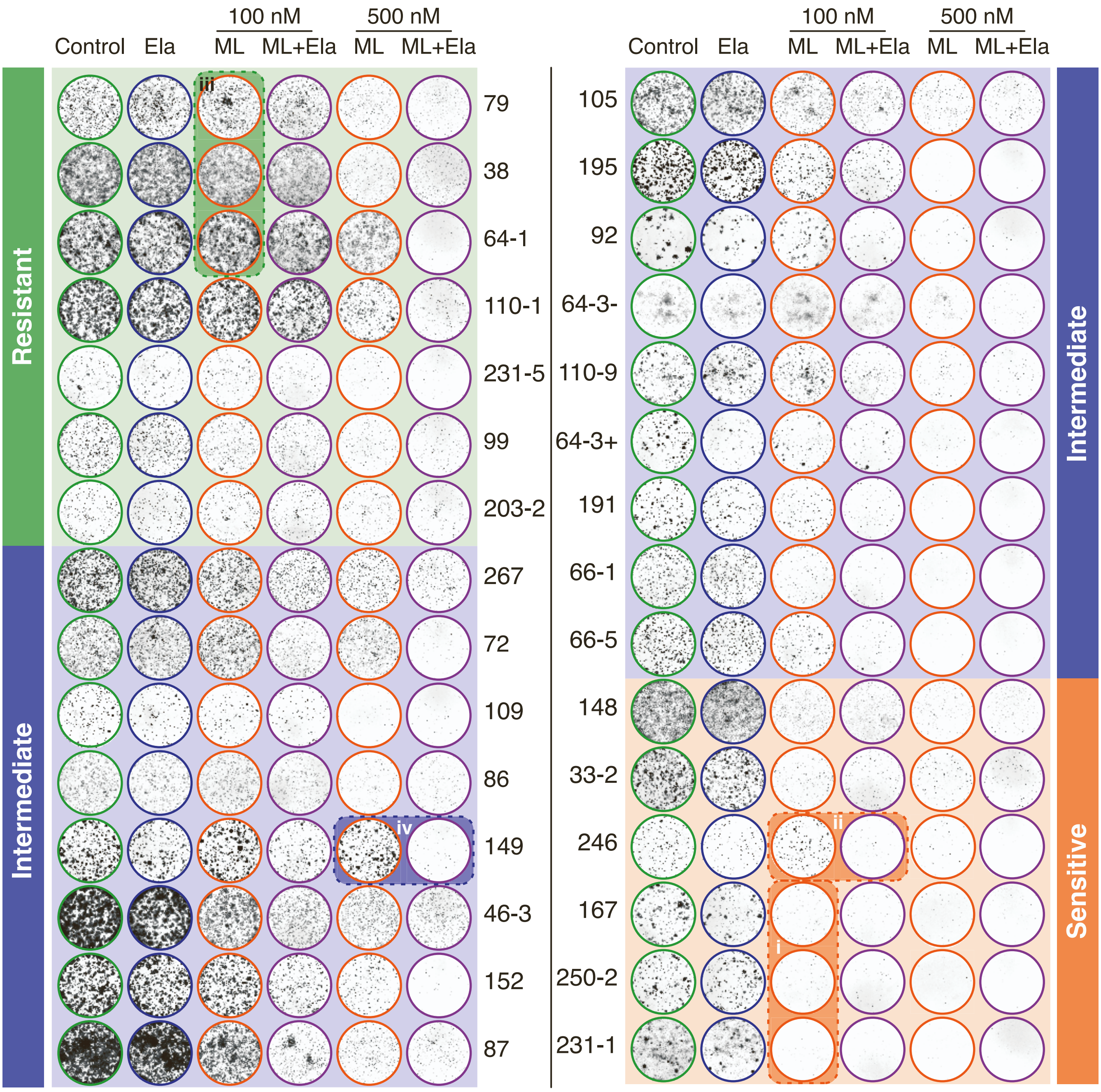

100 nM
500 nM
Control
Ela
ML
ML+Ela
ML
ML+Ela
Resistant
Intermediate
Sensitive
iii
iv
ii
i
79
38
64-1
110-1
231-5
99
203-2
267
72
109
86
149
46-3
152
87
105
195
92
64-3-
110-9
64-3+
191
66-1
66-5
148
33-2
246
167
250-2
231-1

- Figure S5 -

Figure S5. Screen 2: inhibiting drug efflux pump redefines ML-792 sensitivity. (**a**) Colony formation assay images for 30 OCMs treated with DMSO (Control), 250 nM elacridar (Ela), 100 nM ML-792 ± elacridar and 500 nM ML-792 ± elacridar. Categories are based on 100 nM ML-792 plus elacridar CFA values as shown in **Figure 5d**. Panels i–iv highlight exemplars described in the text. Related to **Figure 5**.

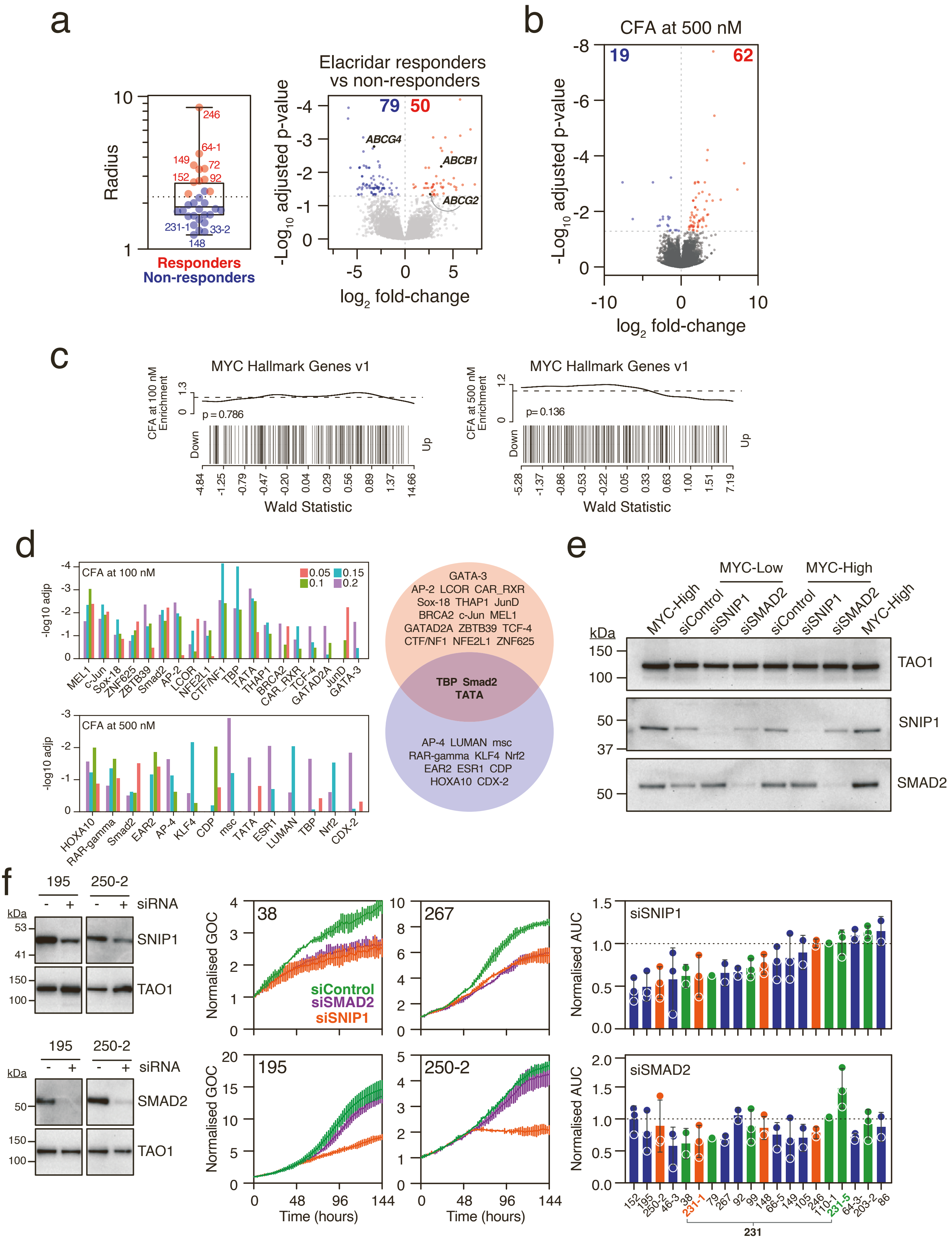

a
Radius
Responders
Non-responders
Elacridar responders vs non-responders
79
50
ABCG4
ABCB1
ABCG2
log2 fold-change
-Log10 adjusted p-value
b
CFA at 500 nM
19
62
c
MYC Hallmark Genes v1
CFA at 100 nM Enrichment
p = 0.786
CFA at 500 nM Enrichment
p= 0.136
Down
Up
Wald Statistic
d
CFA at 100 nM
CFA at 500 nM
-log10 adjp
GATA-3
AP-2 LCOR CAR_RXR
Sox-18 THAP1 JunD
BRCA2 c-Jun MEL1
GATAD2A ZBTB39 TCF-4
CTF/NF1 NFE2L1 ZNF625
TBP Smad2
TATA
AP-4 LUMAN msc
RAR-gamma KLF4 Nrf2
EAR2 ESR1 CDP
HOXA10 CDX-2
e
MYC-Low
MYC-High
kDa
siControl
siSNIP1
siSMAD2
TAO1
SNIP1
SMAD2
f
siRNA
Normalised GOC
siControl
siSMAD2
siSNIP1
Time (hours)
siSNIP1
siSMAD2
Normalised AUC
231

- Figure S6 -

Figure S6. OCMs harbour multiple, non-overlapping vulnerabilities. (**a**) (*Left*) Box-and-whiskers plot showing the distance to origin (radius) for each OCM in **Figure 5c**. Colours indicate the 10 significant elacridar responders (**red**) and the non-responders (**blue**). (*Right*) Volcano plot of differentially expressed genes identified by comparing transcriptomes of elacridar responders versus non-responders, highlighting *ABCB1* and *ABCG2* in the subset of 50 upregulated genes. (**b**) Volcano plot showing differentially expressed genes using colony formation area (CFA) z-scores of OCMs treated with 500 nM ML-792 plus elacridar as a continuous variable, highlighting 19 downregulated and 62 upregulated genes associated with increasing resistance to ML-792. (**c**) Enrichment of Hallmark MYC targets V1 using z-scores from CFA at 100 nM ML-792 ($p=0.786$) or 500 nM ML-792 ($p=0.136$) in the presence of elacridar. (**d**) Differentially expressed genes defined by DESeq2 using CFA values as a continuous variable, at 100 nM (*top*) and 500 nM (*bottom*) ML-792 treatment in the presence of elacridar, identified across four adjusted p-value thresholds (adjusted $p < 0.05$, **orange**; 0.1, **green**; 0.15, **cyan**; 0.2, **purple**). Transcription factor enrichment analysis was conducted using gprofiler2, and transcription factors with a $p \leq 0.05$ were retained and plotted on the x-axis. The y-axis represents the -log10 adjusted p-value for each transcription factor, indicating the strength of enrichment. (*right*) Venn diagram comparing transcription factors identified from the 100 nM ML-792 gene lists (**red**) and the 500 nM ML-792 gene lists (**purple**), revealing three shared transcription factors: TBP, SMAD2, and TATA-binding protein. (**e**) Immunoblots confirming repression of SNIP1 and SMAD2 96 hours after RNAi transfection of FC-MYC cells in the absence (MYC-Low) or presence (MYC-High) of 500 ng/ml tetracycline. TAO1 used as a loading control. (**f**) (*Left*) Immunoblot confirming repression of SNIP1 and SMAD2 by RNAi transfection for 72 hours. TAO1 used as a loading control. (*Middle*) Exemplar 144-hour, GFP-H2B-based proliferation curves used to generate AUC values in (**6d**), with green object count (GOC) determined by time-lapse microscopy and normalised to the T_0 value. Mean ± SD for two technical replicates. (*Right*) Bar graphs showing 20 OCMs rank ordered by sensitivity to siSNIP1, determined by AUC values derived from 144-hour GFP-H2B-based proliferation assays of cells exposed siSNIP1 or siSMAD2 and normalised to non-targeting RNAi. Colours represent ML-792 sensitivity classifications from **Figure 5d**. Data are mean ± SD from three biological replicates, except 79 and 110-1 which are from 2 technical replicates. Related to **Figures 5** and **6** and **Table S3**.

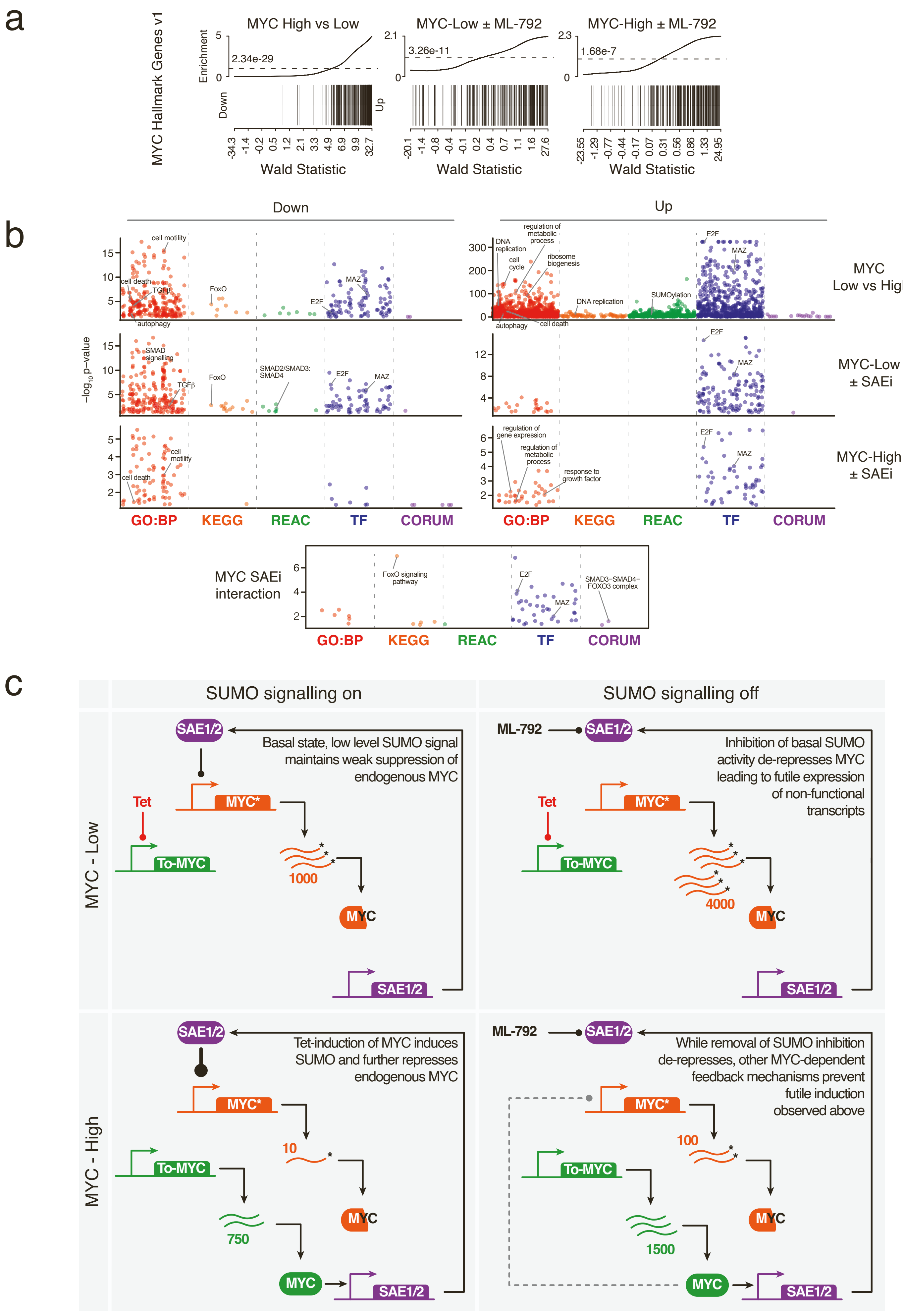
a
MYC Hallmark Genes v1
MYC High vs Low
MYC-Low ± ML-792
MYC-High ± ML-792
Enrichment
2.34e-29
3.26e-11
1.68e-7
Down
Up
Wald Statistic
b
Down
Up
MYC
Low vs High
MYC-Low
± SAEi
MYC-High
± SAEi
$-\log_{10}$ p-value
cell motility
cell death
TGFβ
autophagy
FoxO
E2F
MAZ
DNA replication
regulation of metabolic process
cell cycle
ribosome biogenesis
SUMOylation
SMAD signalling
SMAD2/SMAD3:SMAD4
regulation of gene expression
response to growth factor
GO:BP
KEGG
REAC
TF
CORUM
MYC SAEi interaction
FoxO signaling pathway
SMAD3-SMAD4-FOXO3 complex
c
SUMO signalling on
SUMO signalling off
MYC - Low
MYC - High
SAE1/2
ML-792
Basal state, low level SUMO signal maintains weak suppression of endogenous MYC
Inhibition of basal SUMO activity de-represses MYC leading to futile expression of non-functional transcripts
Tet-induction of MYC induces SUMO and further represses endogenous MYC
While removal of SUMO inhibition de-represses, other MYC-dependent feedback mechanisms prevent futile induction observed above
Tet
MYC*
To-MYC
MYC
1000
4000
10
100
750
1500

- Figure S7 -

Figure S7. SUMO signalling inhibits transcription of MYC. (**a**) Gene set enrichment analysis of MYC Hallmark Genes V1 comparing MYC-High versus MYC-Low, or ± 25 nM ML-792 in MYC-Low and MYC-High cells, showing p values. (**b**) Pathway analysis of downregulated (Down) and upregulated (Up) genes in the conditions described for (**a**), and the 132 genes identified as behaving differently upon exposure to ML-792 depending on MYC level (interaction). (**c**) Model to explain how ML-792-mediated inhibition of SUMO signalling in MYC-Low cells de-represses the endogenous MYC alleles leading to expression of non-functional transcripts, marked by an asterisk. In the absence of SUMO signalling, induction of transgenic MYC activates alternative negative autoregulation mechanisms to limit expression of endogenous MYC. Numbers approximating transcript abundance are based on data shown in **Figure 7e** (right panel). Related to **Figure 7**.

Patient					OCM							
#	ID	FIGO Stage	Subtype[a]	*TP53* Primary tumour (targeted amplicon)	OCM ID[b]	Chemo -naïve (Y/N)[c]	Biopsy type	*TP53* (sequencing of cloned transcripts) DNA	*TP53* (sequencing of cloned transcripts) Protein prediction	p53 immunostaining +/- Nutlin-3: -Nutlin-3[d]	p53 immunostaining +/- Nutlin-3: +Nutlin-3[e]	References
1	33	4B	HGSOC	NA	33-2	N	Ascites	c.783insATT	Inframe insertion	+	-	[S2], [S3], [S4], [S5]
2	38	3C	HGSOC	c.376-1G>C	38	Y	Ascites	c.375_395del	Inframe deletion	-	-	[S1], [S2], [S3], [S5]
3	46	3C	HGSOC	c.267delC	46-3	N	Ascites	c.267delC	Frameshift	-	-	[S2], [S3], [S4], [S5]
4	64	3C	Possible mixed LGSOC/HGSOC[†]	c.646G>A	64-1	N	Ascites	c.646G>A	p.V216M	+	-	[S2], [S3], [S4], [S5]
					64-3-Ep-	N	Ascites			+	-	[S2], [S3], [S5]
					64-3-Ep+					+	-	[S2], [S3]
5	66	3C	HGSOC	Neoplastic cell count less than 10%	66-1	N	Ascites	c.488A>G	p.Y163C	+	-	[S2], [S3], [S4], [S5]
					66-5	N	Ascites	c.488A>G	p.Y163C	+	-	[S2], [S3], [S5]
6	72	1A	Moderately differentiated MOC	c.843C>G	72	N	Ascites	c.843C>G	p.D281E	+	-	[S2], [S3], [S5]
7	79	3C	HGSOC	NA	79	N	Ascites	c.153_162del	Frameshift	-	-	[S2], [S3], [S5]
8	86	4B	HGSOC	NA	86	N	Ascites	c.842A>G	p.D281G	-	-	[S3], [S4]
9	87	3B	Possible CCOC[†]	NA	87	Y	Ascites	WT	WT	+	+	[S2], [S3], [S4], [S5]
10	92	3C	HGSOC	WT	92	N	Ascites	WT	WT	-	ND	[S3], [S4]
11	99	3C	HGSOC	Insufficient DNA	99	Y	Ascites	c.810T>G	p.F270L	+	ND	[S3], [S4]
12	105	3C	HGSOC	Insufficient DNA	105	N	Ascites	c.431A>C	p.Q105P	+	-	[S4], [S5]
13	109	4B	HGSOC	Insufficient DNA	109	N	Ascites	c.743G>A	p.R248Q	+	-	[S3], [S4], [S5], [S6]
14	110	3C	HGSOC	ND	110-1	Y	Ascites	c.743G>A	p.R248Q	+	-	[S1], [S3], [S4]
				ND	110-9	N	Ascites	ND	ND	ND	ND	[S3]
15	148	3C	HGSOC	ND	148	N	Ascites	c.713G>A	p.C238Y	+	-	This study
16	149	3C	HGSOC	NA	149	N	Ascites	c.724T>G	p.C242G	+	-	[S3], [S4]
17	152	3C	Moderately differentiated serous adenocarcinoma of intermediate grade[†]	NA	152	N	Ascites	c.659A>G	p.Y220C	+	-	[S3], [S4], [S5]
18	167	3C	HGSOC	c.742C>T[f]	167	N	Ascites	WT	WT	+	ND	[S3], [S4]

19	191	3A	HGSOC	c.743G>A	191	N	Ascites	c.743G>A	p.R248Q	+	ND	[S3], [S4], [S5]
20	195	4A	Possible LGSOC[†]	WT	195	Y	Ascites	WT	WT	+	+	[S3], [S4], [S5]
21	203	3C	HGSOC	c.1025G>C	203-2	N	Ascites	c.1025G>C	p.R342P	+	ND	[S4]
22	231	3C	HGSOC	NA	231-1	Y	Ascites	c.742C>G	p.R248G	+	-	[S3], [S4]
					231-5	N	Ascites	c.742C>T	p.R248W	+	-	This study
23	246	4A	HGSOC	c.309C>G	246	N	Ascites	c.309C>G	p.Y103*	-	ND	[S4], [S6]
24	250	3C	HGSOC	c.584T>A	250-2	N	Ascites	c.584T>A	p.I195N	+	+	[S4]
25	267	4B	HGSOC	NA	267	N	Ascites	c.659A>G	p.Y220C	+	ND	[S4]

Table S1. Patient and OCM characteristics

a, Based on histology and other information where indicated by † (see Barnes *et al*. 2021).

b, The models are referred to using the OCM prefix followed by the patient number and, if one of a longitudinal series, the biopsy number. Models generated independently from the same biopsy are distinguished by an alphabetical suffix.

c, At the time of the research biopsy.

d, p53 status without Nutlin-3 treatment: '-' indicates p53 not detected, '+' indicates p53 detected.

e, p53 status following Nutlin-3 treatment: '-' indicates no induction of p53, '+' indicates induction of p53.

f, This *TP53* variant was detected at a variant allele frequency of 6% in a tumour block with >50% tumour cell content.

CCOC, clear cell ovarian cancer; fs, frameshift; HGSOC, high-grade serous ovarian cancer; LGSOC, low-grade serous ovarian cancer; MOC, mucinous ovarian cancer; NA, not available; ND, not done; WT, wildtype.

Supplemental References: [**S1**] Pillay, N., *et al*. (2019) DNA Replication Vulnerabilities Render Ovarian Cancer Cells Sensitive to Poly(ADP-Ribose) Glycohydrolase Inhibitors. Cancer Cell, 35, 519-533 e518. [**S2**] Nelson, L., Tighe, A., *et al*. (2020) A living biobank of ovarian cancer *ex vivo* models reveals profound mitotic heterogeneity. Nat Commun, 11, 822. [**S3**] Barnes, B.M., *et al*. (2021) Distinct transcriptional programs stratify ovarian cancer cell lines into the five major histological subtypes. Genome Med, 13, 140. [**S4**] Coulson-Gilmer, C., *et al*. (2021) Replication catastrophe is responsible for intrinsic PAR glycohydrolase inhibitor-sensitivity in patient-derived ovarian cancer models. J Exp Clin Cancer Res, 40, 323. [**S5**] Golder, A., *et al*. (2022) Multiple-low-dose therapy: effective killing of high-grade serous ovarian cancer cells with ATR and CHK1 inhibitors. NAR Cancer, 4, zcac036. [**S6**] Coulson-Gilmer, C., *et al*. (2024) Intrinsic PARG inhibitor sensitivity is mimicked by *TIMELESS* haploinsufficiency and rescued by nucleoside supplementation. NAR Cancer, 6. zcae030.
